# Computer-based assessment of unilateral spatial neglect: A systematic review

**DOI:** 10.3389/fnins.2022.912626

**Published:** 2022-08-19

**Authors:** Ioanna Giannakou, Dan Lin, David Punt

**Affiliations:** ^1^School of Sport, Exercise and Rehabilitation Sciences, University of Birmingham, Birmingham, United Kingdom; ^2^School of Health & Life Sciences, Teesside University, Middlesbrough, United Kingdom

**Keywords:** attention, unilateral spatial neglect (USN), computer-based assessment, stroke, visual search, systematic review

## Abstract

**Background:**

To date, no gold standard exists for the assessment of unilateral spatial neglect (USN), a common post-stroke cognitive impairment, with limited sensitivity provided by currently used clinical assessments. Extensive research has shown that computer-based (CB) assessment can be more sensitive, but these have not been adopted by stroke services yet.

**Objective:**

We conducted a systematic review providing an overview of existing CB tests for USN to identify knowledge gaps and positive/negative aspects of different methods. This review also investigated the benefits and barriers of introducing CB assessment tasks to clinical settings and explored practical implications for optimizing future designs.

**Methodology:**

We included studies that investigated the efficacy of CB neglect assessment tasks compared to conventional methods in detecting USN for adults with brain damage. Study identification was conducted through electronic database searches (e.g., Scopus), using keywords and standardized terms combinations, without date limitation (last search: 08/06/2022). Literature review and study selection were based on prespecified inclusion criteria. The quality of studies was assessed with the quality assessment of diagnostic accuracy studies tool (Quadas-2). Data synthesis included a narrative synthesis, a table summarizing the evidence, and vote counting analysis based on a direction of effect plot.

**Results:**

A total of 28 studies met the eligibility criteria and were included in the review. According to our results, 13/28 studies explored CB versions of conventional tasks, 11/28 involved visual search tasks, and 5/28 other types of tasks. The vote counting analysis revealed that 17/28 studies found CB tasks had either equal or higher sensitivity than conventional methods and positive correlation with conventional methods (15/28 studies). Finally, 20/28 studies showed CB tasks effectively detected patients with USN within different patient groups and control groups (17/28).

**Conclusions:**

The findings of this review provide practical implications for the implementation of CB assessment in the future, offering important information to enhance a variety of methodological issues. The study adds to our understanding of using CB tasks for USN assessment, exploring their efficacy and benefits compared to conventional methods, and considers their adoption in clinical environments.

## Introduction

Unilateral spatial neglect (USN) is one of the most common post-stroke cognitive impairments with a prevalence of up to 80% early after stroke (Stone et al., 1993; Hammerbeck et al., 2019) and around 30% in the chronic phase of stroke (Esposito et al., 2021). USN as defined by Heilman et al. (1987) is an inability to explore or respond to a stimulus on the contralesional side of space, provided that this failure is not caused by lower-level sensory, motor, or visual impairment. USN can be observed either on the left or right side of space with higher frequency and more severe lateralized attention deficits on the left side (Ten Brink et al., 2017). In the early stages of stroke, the severity of USN can be a prognostic factor of increased hospital length of stay, worse rehabilitation outcome, family burden, and long-lasting impairments (Buxbaum et al., 2004; Luvizutto et al., 2018; Hammerbeck et al., 2019). The severity of neglect has also been associated with a higher risk of falls, reduced quality of life, reduced functional outcome, and reduced independence in the chronic stages (Jehkonen et al., 2006; Chen et al., 2015). The underlying mechanisms of USN have been intensively investigated by researchers, with proposed theories mainly focused on representational (Bisiach and Luzzatti, 1978; Milner et al., 1993) and attentional factors (Heilman and Van Den Abell, 1980; Posner et al., 1982; Kinsbourne, 1993). However, some aspects of the theoretical underpinnings of USN remain controversial in the literature (Karnath and Rorden, 2012; Baldassarre et al., 2014; Montedoro et al., 2018), possibly as a result of the complex and heterogeneous nature of the neglect syndrome; and diverse subtypes might be associated with varied brain areas and forms of the disorder (Rode et al., 2017; Gammeri et al., 2020). For example, USN can subdivide based on spatial domains and frames of reference (Buxbaum et al., 2004; Caggiano and Jehkonen, 2018). And in the last 20 years, neuroanatomical studies have highlighted associations with parietal, temporal, and frontal lobe brain lesions in affected patients (Corbetta and Shulman, 2011; Lunven and Bartolomeo, 2017; Zebhauser et al., 2019).

An additional challenge in our understanding of USN is diagnosis, associated with both the lack of gold standard assessment and the use of numerous and varied diagnostic tools (Azouvi et al., 2016). Menon and Korner-Bitensky (2004) detected more than 60 behavioral tests and functional assessment tools with a large variety of apparatus use, task requirements/design, and diagnostic accuracy, also highlighting different neglect subtypes or syndrome components (Verdon et al., 2010; Grattan and Woodbury, 2017). National clinical guidelines for stroke suggest using standardized assessments to assess USN, including paper-and-pencil (PnP) tests (Royal College of Physicians, 2016; Canadian Stroke Best Practice Recommendations, 2019). A multidisciplinary international survey (Checketts et al., 2021) reported that currently the most widely used USN assessments include those such as the Behavioral Inattention Test (BIT), developed by Wilson et al. (1987), and the Catherine Bergego Scale (CBS), developed by Azouvi (1996). Even though these tests are used on a daily basis in clinical practice, there remains criticism regarding how optimal they are, and studies have demonstrated that they still suffer from many limitations related to lack of precision, ecological validity, reduced sensitivity, and high false-negative results (Rengachary et al., 2009; Bonato and Deouell, 2013; Kaufmann et al., 2020).

There remains a need for higher quality assessment methods to be introduced to clinical practice and in the last two decades, a variety of studies have demonstrated that computer-based (CB) tasks may be able to make a significant contribution. These tasks can provide higher sensitivity and diagnostic accuracy (also detecting mild and chronic cases) and better psychometric properties (especially diagnostic validity) with low cost and short administration time (Schendel and Robertson, 2002; Deouell et al., 2005; Erez et al., 2009; Rengachary et al., 2009; Bonato, 2012; Villarreal et al., 2020). These CB tasks appear to be well-accepted by patients, and offer design flexibility, stimulus modification, adjustment of difficulty, and may also limit the use of compensatory strategies by patients. They may also be less likely to include floor and ceiling effects (Bonato et al., 2010, 2013; Bonato, 2012). These tasks can also provide important patient information regarding subtype and severity of neglect providing data to inform a more specific individually tailored rehabilitation program (Ulm et al., 2013; Dalmaijer et al., 2015). Computer-based tasks may therefore provide an opportunity to augment USN assessment in clinical practice; PnP are likely to remain important, due to some practical limitations for some CB tests such as the need for specified hardware or software, and the current requirement for basic programming/statistical skills to implement them (Bonato and Deouell, 2013). However, the practical limitations of CB assessment have been gradually minimized, and the past decade has seen the rapid increase of the accessibility of computers and their role in everyday life (e.g., education, entertainment etc.) providing a more supportive environment for their implementation in clinical practice.

This review draws a distinction between CB assessment and assessment using virtual reality (VR). Previous reviews have focused on VR -tasks (immersive and non-immersive) used in the assessment and rehabilitation of USN, demonstrating that VR assessment can effectively detect USN (Tsirlin et al., 2009; Pedroli et al., 2015; Ogourtsova et al., 2017). In contrast, this is the first study to provide an evaluation and overview of existing CB assessment tools for USN, not involving VR. The reviewers were not able to find existing operational definitions in the related literature for CB assessment and how this differs from non-immersive VR tasks; however, there is a clear distinction, since there was only very minimal overlap in this review (1 out of 28 studies) with a VR-focused systematic review (Ogourtsova et al., 2017). For the purpose of this systematic review, CB tasks were defined as screen-based tasks, where the authors of included studies defined these as CB tasks (even if they could potentially be taxonomized in the gray area of CB tasks and non-immersive VR tasks), using a variety of different displays (e.g., monitor, projector, tablet).

This systematic review will shed light on the methodological inconsistencies in related studies and provide an evaluation and overview of the existing evidence around the CB assessment of USN. The results will offer future researchers essential background knowledge for designing and optimizing CB tasks. This review will also explore the advantages and challenges for stroke services in adopting CB assessment for USN. To our knowledge, no previous study has conducted a systematic review on CB assessment for USN. To define the review question, a PICO (population, intervention, comparison, and outcome) framework was adopted (Higgins et al., 2019).

“Do CB tasks enhance the ability to detect USN in stroke survivors compared with conventional tests?”

PICO:

Participants: Studies including adult (aged over 18 years) stroke survivors or patients with other types of brain damage with or without USN.

Intervention: CB tasks (screen-based tasks that are categorized by the authors of the included studies as CB assessment/testing or computerized tasks etc., using displays such as monitor, projector, tablet etc.).

Comparison: Conventional tests (e.g., PnP, or functional assessment) or CB design based on PnP.

Outcomes: A variety of outcomes were accepted such as [diagnostic accuracy measures (e.g., sensitivity, specificity), psychometric properties (e.g., validity, reliability), correlation coefficient, subjects' performance data, narrative reports].

## Methodology

The review was conducted based on the preferred reporting items for systematic reviews and meta-analysis (PRISMA) guidelines (Moher et al., 2009).

### Search strategy

This review considered studies focusing on evaluating the diagnostic accuracy and performance of CB tasks in identifying USN in stroke survivors and patients with other types of brain damage.

A comprehensive search strategy of electronic databases (PsycINFO, EMBASE, MEDLINE (OVID), AMED, CINAHL (EBSCO), WEB of Science, PubMed, Scopus) using keywords and a combination of standardized terms was conducted.

The last search was conducted on 08/06/2022 with the following search strategy keywords:

- (“brain damage” OR “brain lesion” OR aneurysm OR “transient ischaemic attack” OR “ischemic attack” OR TIA OR “cerebrovascular accident” OR “cerebral vascular accident” OR CVA OR “traumatic brain injury” OR TBI OR stroke OR “brain tumor”) AND
- (neglect OR “visuospatial neglect” OR inattention OR “unilateral neglect” OR “unilateral inattention” OR hemineglect OR “unilateral spatial neglect” OR “hemispatial neglect” OR “visual neglect” OR “hemi-inattention” OR “perceptual disorder^*^”) AND
- (assess^*^ OR measur^*^ OR evaluat^*^ OR test^*^ OR screen^*^ OR diagnos^*^ OR assessment OR measurement OR evaluation OR diagnosis OR screening) AND
- (computer^*^ OR computer OR computerized OR computer-based OR phone OR smartphone OR tablet)

In order to retrieve further published, unpublished and ongoing studies, manual searches were utilized through the references of included articles, gray literature was identified through Google Scholar and registered ongoing trials were searched [ClinicalTrials.gov (http://clinicaltrials.gov/)].

No restriction was considered regarding the publication date or status. Studies were not limited to those written in English, but all eligible studies were written in English.

### Eligibility criteria

- Inclusion criteria

- Context: studies that present or evaluate the diagnostic accuracy and performance of CB tests for USN in stroke survivors and patients with other types of brain damage (e.g., brain lesion, aneurysm, traumatic brain injury, or brain tumor etc.).
- Studies that fulfill the criteria established by the PICO framework.
- The term “CB tasks” was not consistent in the literature, so the reviewers decided to include studies exploring screen-based tasks presented by the authors of the included studies as CB assessment/testing (or using related synonyms such as computerized or digitized/computer version of PnP etc.). We included CB tasks that followed these criteria even if they could be potentially classified in the gray area of CB tasks and non-immersive VR tasks, using a variety of different apparatus (e.g., computer-monitor, projectors, tablet).

- Exclusion criteria

- Studies not including stroke survivors or patients with other types of brain damage.
- Studies that did not focus on USN assessment.
- Studies where CB task is not the index test.
- Studies where the design of the CB task and apparatus are not substantially defined so that the reviewers can decide whether it can be included in the review. Studies that did not clarify the type of apparatus (e.g., response box, computer-monitor, projector) or specific design details (e.g., task demands and outcome measures) were excluded.
- Studies that did not clarify the use of a comparison tool.
- Studies that did not report or evaluate any form of diagnostic accuracy or performance of the CB test.
- Studies exploring VR based tasks (as defined by the authors and/or as interpreted by the reviewers).

### Data extraction and selection

Duplicates were removed and IG screened the title and abstract of all identified studies, excluding irrelevant studies. The remaining studies were screened by full-text and excluded based on the eligibility criteria. This was verified by DL and in the case of conflict, it was resolved by discussion and if the discrepancy was not resolved, a third author DP would be included (but it was not needed).

IG and DL performed data extraction using a data extraction sheet (Supplementary Table 1), that was developed to accurately collect study characteristics and data. Data collection was verified, and disagreements resolved by discussion and consensus.

Extracted data included:

- Study Characteristics: study type, population, sample size, year, number of participants with USN (+) and without USN (-), patients with right brain damage (RBD) or left-brain damage (LBD), follow-up, inclusion/exclusion criteria, etc.
- CB task information (e.g., apparatus, outcome measures, administration time) and reference standards comparison for diagnosing USN.
- Study data: diagnostic accuracy measures, psychometric properties, outcome measures efficacy, patient history data, and test performance data, etc.
- Information for risk of bias assessment.

### Risk of bias (quality) assessment

IG and DL independently used the quality assessment tool for diagnostic accuracy studies (QUADAS-2) for systematic reviews to evaluate the quality of evidence for the identified studies (Whiting et al., 2011).

### Data synthesis

**Figure 1:**
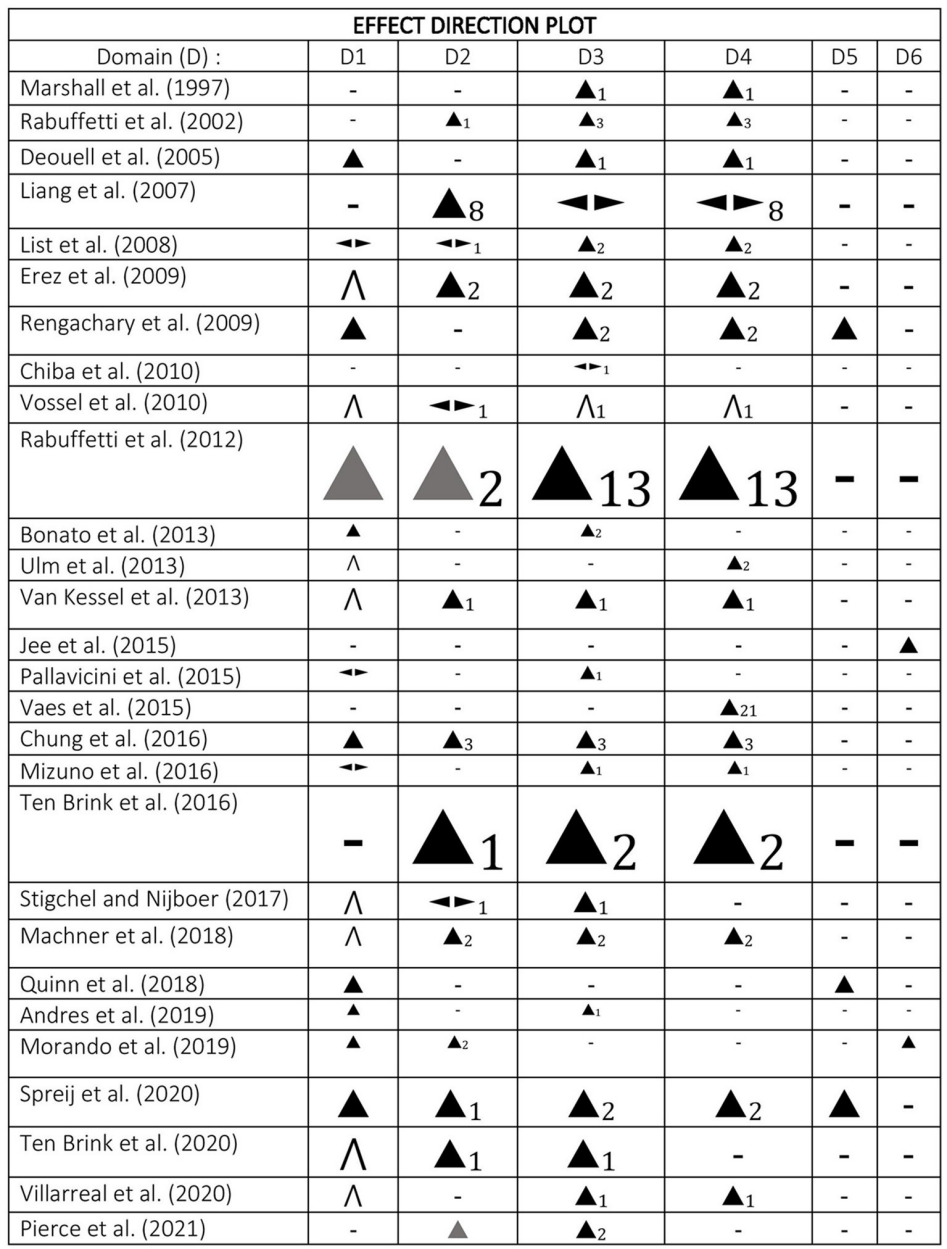
Effect direction plot.

This review includes a narrative synthesis and analysis of the results of the included studies (McKenzie and Brennan, 2019). Studies were tabulated and compared in groups based on the index test type (e.g., CB version of PnP, visual search tasks, and other types) and heterogeneity was explored according to the type of analysis, data, and index test (Campbell et al., 2020). Study characteristics were synthesized and presented in a table (Supplementary Table 2) containing summaries of the outcome measures, patient history data, study details, and risk of bias. The main results were tabulated (Supplementary Table 3) and data were analyzed and presented as an effect direction plot (Figure 1) for 6 domains (sensitivity, correlation coefficient with conventional task or neglect symptoms, ability to distinguish among patient groups, ability to distinguish patients from control groups, specificity, reliability) (Boon and Thomson, 2021). In order to gain a better understanding and deeper analysis of the data, a vote counting technique of effect direction was conducted (McKenzie and Brennan, 2019).

Ideally, we would have performed a meta-analysis of our dataset from the included studies to compare outcomes of the CB assessment tools including properties (e.g., sensitivity, specificity) and outcome measures (e.g., reaction times (RT) and accuracy). However, there was not sufficient data with acceptable homogeneity to undertake a meta-analysis (Deeks et al., 2019).

## Results

**Figure 2:**
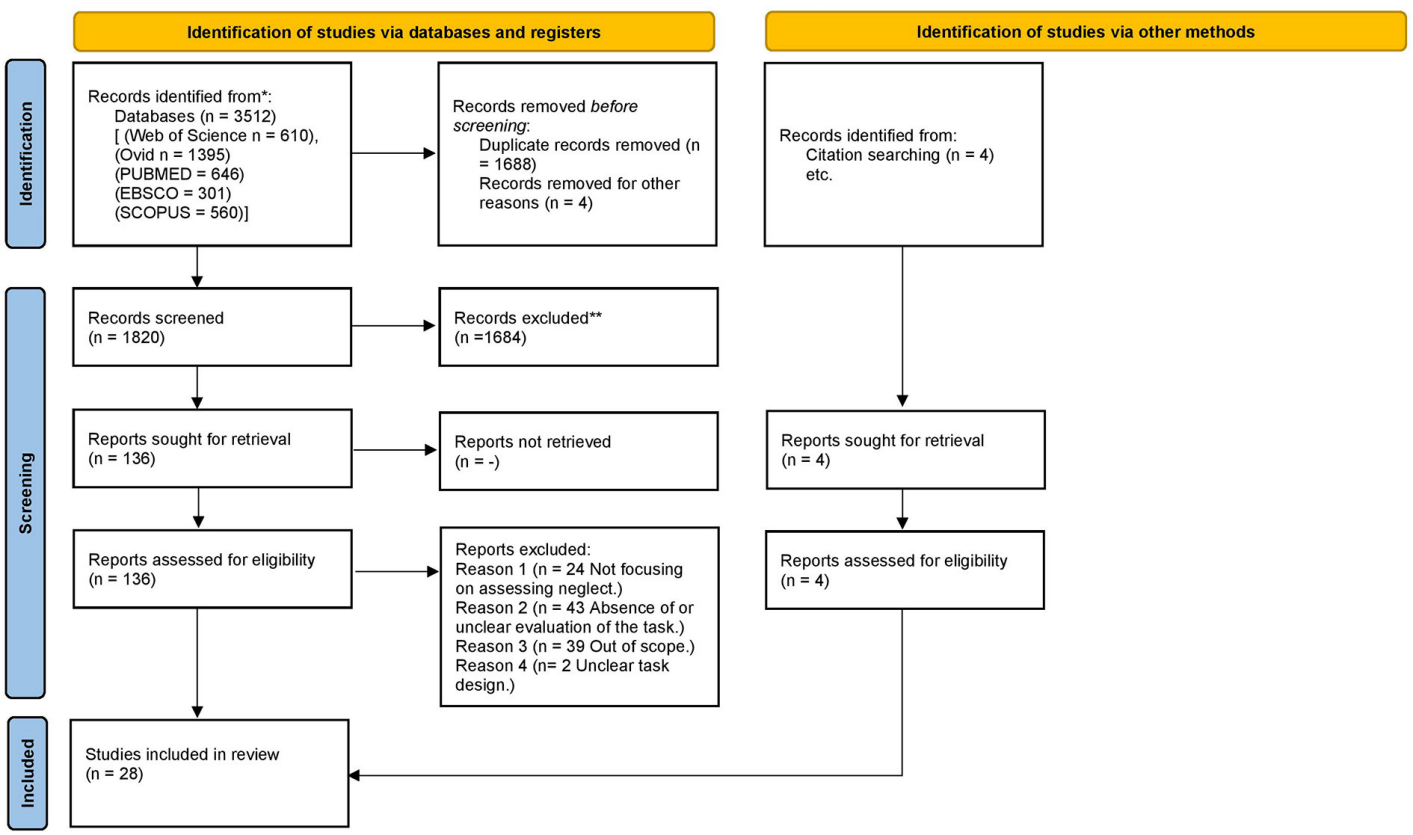
PRISMA flow diagram.

In the current systematic review, we aimed to provide a review of studies evaluating CB assessment of USN. The screening process is summarized within a PRISMA flow diagram (Figure 2) (Page et al., 2021). In total, 28 articles met the inclusion criteria and were critically appraised. However, we full screened 136 studies. The excluded citations and the reasons for exclusion were tabulated (Supplementary Table 4). There were three main reasons for the exclusion of studies that were not out of scope. The first case was where there was either an absence or not clear use of a comparison tool. Secondly, studies were excluded where authors did not clarify whether USN was assessed. Lastly, studies were excluded when the specific details regarding the design of the CB tasks were not explained thoroughly enough to allow the reviewers to decide whether the study was eligible for inclusion. This process led to excluding some important studies that explored CB assessment methods by using overlays of PnP on graphic tablets (Donnelly et al., 1999; Guest et al., 2002), CB versions of PnP tests (Halligan and Marshall, 1989; Kerkhoff and Marquardt, 1995; Chiba et al., 2006; Smit et al., 2013; Van der Stoep et al., 2013; Hopfner et al., 2015) and software for analysis (Rorden and Karnath, 2010; Dalmaijer et al., 2015). Moreover, the reviewers had to exclude a large body of work that investigated target/stimulus detection tasks (Beis et al., 1994; Tipper and Behrmann, 1996; Baylis et al., 2004), RT tasks (Anderson et al., 2000; Schendel and Robertson, 2002; Sacher et al., 2004), visual search task (Laeng et al., 2002; Toba et al., 2018; Borsotti et al., 2020), visual attention theory focused tasks (Habekost and Bundesen, 2003; Bublak et al., 2005) and the test battery of attentional performance (TAP) (Zimmermann and Fimm, 2002). Supplementary Table 2 illustrates some of the main characteristics of the included studies (risk of bias, subjects, conventional tool comparison, task details, results, and conclusions). The studies were grouped based on the CB assessment type in three groups [1. CB versions of conventional tasks, 2. Visual search tasks (2a. Dynamic & dual tasks, 2b. Feature and conjunction tasks, 2c. Static tasks) 3. Different types of tasks]. In our synthesis, we explored the apparatus, response method, outcome measure, comparison, and properties of the CB tools. Supplementary Table 3 represents a summary of the main results of the studies and Figure 1 is an effect direction plot based on the vote counting analysis conducted.

### Computer-based task type

The authors identified 13 studies, that investigated test batteries of CB versions of conventional tasks, including tasks similar to line bisection (Chiba et al., 2010; Jee et al., 2015), cancellation (Rabuffetti et al., 2002, 2012), baking tray (Chung et al., 2016) or combinations of different types of tasks (Liang et al., 2007; Ulm et al., 2013; Pallavicini et al., 2015; Vaes et al., 2015; Mizuno et al., 2016; Ten Brink et al., 2016; Quinn et al., 2018; Morando et al., 2019). Our synthesis included 11 studies exploring visual search tasks such as static (Mizuno et al., 2016; Machner et al., 2018; Ten Brink et al., 2020), feature and conjunction (List et al., 2008; Erez et al., 2009), dynamic and dual tasks (Marshall et al., 1997; Deouell et al., 2005; Bonato et al., 2013; Van Kessel et al., 2013; Andres et al., 2019; Villarreal et al., 2020). We detected five studies that observed different types of tasks such as the widely investigated Posner cueing paradigm (Rengachary et al., 2009), a neglect/extinction task (Vossel et al., 2010), a temporal order judgment (TOJ) task (Stigchel and Nijboer, 2017), a driving simulator task (Spreij et al., 2020) and a manual spatial exploration task (Pierce et al., 2021).

By carefully examining the data, it was found that CB functional (Pallavicini et al., 2015) and visual search tasks were shown to be more sensitive than CB versions of PnP tasks (Ten Brink et al., 2020). Moreover, combining different types of tasks can capture a wider aspect of neglect symptoms (Erez et al., 2009; Mizuno et al., 2016; Spreij et al., 2020). An interesting finding was that different types of tasks were associated with different types of errors, for example, cancellation tasks were more sensitive to viewer-centered (egocentric) errors, with visual search and line bisection tasks more sensitive to stimulus-centered (allocentric) errors; Similarly RT and TOJ tasks also detected spatial bias deficits (Van Kessel et al., 2013; Mizuno et al., 2016; Stigchel and Nijboer, 2017).

Some studies compared diagnostic accuracy between CB tests, and as expected the more complex CB tasks (e.g., higher demands, dual tasks, increased number of targets, conjunction tasks) were more sensitive and could detect more cases (chronic/subclinical) than more simple versions (e.g., feature tasks) (Marshall et al., 1997; List et al., 2008; Erez et al., 2009; Bonato et al., 2013; Van Kessel et al., 2013; Andres et al., 2019; Ten Brink et al., 2020; Villarreal et al., 2020).

### Outcome measures

Most cancellation tasks obtained measures including the number of touched (canceled) targets, revisits, intersections, omissions, center of cancellation (CoC) (Rabuffetti et al., 2002, 2012; Ulm et al., 2013; Pallavicini et al., 2015; Ten Brink et al., 2016). Line bisection tasks usually captured the mean deviation (Chiba et al., 2010; Ulm et al., 2013; Jee et al., 2015).

Visual search and exploration tasks included measures based on false alarms/catch trials responses, target detection (capturing accuracy, detection rate/probability) and time (such as RT, search time and task duration); data analysis in these tasks was performed based on target position [e.g., left/right (L/R)] and task demands (e.g., level of difficulty, number of distractors) (Marshall et al., 1997; Deouell et al., 2005; List et al., 2008; Erez et al., 2009; Vossel et al., 2010; Rengachary et al., 2011; Bonato et al., 2013; Van Kessel et al., 2013; Vaes et al., 2015; Mizuno et al., 2016; Machner et al., 2018; Andres et al., 2019; Ten Brink et al., 2020; Villarreal et al., 2020).

In the course of this work, we discovered that (L/R) target detection (Bonato et al., 2013; Van Kessel et al., 2013; Pallavicini et al., 2015; Machner et al., 2018; Andres et al., 2019), hit rate (response rate) (Erez et al., 2009; Ten Brink et al., 2020), RT asymmetry scores (Deouell et al., 2005; Rengachary et al., 2009; Van Kessel et al., 2013; Machner et al., 2018) and the number of intersection (disorganized search) (Ten Brink et al., 2016) were all shown to be among the most sensitive measures for spatial bias and visual search deficits detection in patients with brain damage.

### Apparatus and response type

A number of studies have used manual response displays such as touchscreen monitors (Rabuffetti et al., 2002, 2012; Ulm et al., 2013; Ten Brink et al., 2016, 2020), smartphone/tablet devices (Pallavicini et al., 2015; Chung et al., 2016; Quinn et al., 2018; Morando et al., 2019; Pierce et al., 2021) or graphic tablets (Liang et al., 2007; Vaes et al., 2015). Several authors have explored the efficacy of using projectors/large screens (Van Kessel et al., 2013; Machner et al., 2018; Spreij et al., 2020; Villarreal et al., 2020), PC/laptop with a mouse (Marshall et al., 1997) or with a response box (Deouell et al., 2005; Erez et al., 2009; Rengachary et al., 2009; Chiba et al., 2010; Vossel et al., 2010; Stigchel and Nijboer, 2017). Some tests are designed requiring verbal (List et al., 2008; Bonato et al., 2013; Andres et al., 2019) or both verbal and manual responses (Chiba et al., 2010). Various tasks captured performance with more than one type of response or apparatus (Van Kessel et al., 2013; Jee et al., 2015; Vaes et al., 2015).

Some methods may be more accurate than others, however, the literature was reviewed, and no apparatus or response type was proved to be superior to any other.

### Specific details

Tasks could take from either 5 to 10 min to complete (Marshall et al., 1997; Van Kessel et al., 2013) or 10–20 min (List et al., 2008; Rengachary et al., 2009; Vossel et al., 2010; Ulm et al., 2013) and test batteries could last more than 20 min (Vaes et al., 2015). The CB tasks were considered to provide accurate results within short administration time (Ulm et al., 2013; Pierce et al., 2021). Authors suggest they also offer reduced overall assessment time compared to conventional methods (e.g., batteries of pen and paper tasks), which is important as long administration time can cause fatigue to the patients and modulate the results (Liang et al., 2007; List et al., 2008).

The diameter of the screen/display varied from 13–15 inches (Deouell et al., 2005; Chiba et al., 2010; Ten Brink et al., 2020) to 17–19 inches (Rengachary et al., 2009; Rabuffetti et al., 2012; Andres et al., 2019) to 24–33 inches (Mizuno et al., 2016; Machner et al., 2018). The distance from the subject could be around 30–46 cm (Chiba et al., 2010; Mizuno et al., 2016; Quinn et al., 2018), 60–70 cm (List et al., 2008; Andres et al., 2019) or 90–100 cm (Deouell et al., 2005; Stigchel and Nijboer, 2017). These two factors were not found to affect the tasks' accuracy or the participants' performance.

### Sensitivity

Sensitivity was reported in 20/28 studies with four reporting in the form of a percentage (Bonato et al., 2013; Chung et al., 2016; Quinn et al., 2018; Spreij et al., 2020), four with statistical significance values (Deouell et al., 2005; Rengachary et al., 2009; Rabuffetti et al., 2012; Andres et al., 2019) and 11 in a narrative manner (List et al., 2008; Erez et al., 2009; Vossel et al., 2010; Ulm et al., 2013; Van Kessel et al., 2013; Pallavicini et al., 2015; Mizuno et al., 2016; Stigchel and Nijboer, 2017; Machner et al., 2018; Ten Brink et al., 2020; Villarreal et al., 2020). Some CB tools had varied results, reporting both positive and negative or neutral outcomes (List et al., 2008; Pallavicini et al., 2015; Mizuno et al., 2016). Overall 17/28 CB tasks report superior or equal sensitivity in detecting USN symptoms when compared to a variety of conventional tasks (Deouell et al., 2005; Erez et al., 2009; Vossel et al., 2010; Rabuffetti et al., 2012; Ulm et al., 2013; Van Kessel et al., 2013; Chung et al., 2016; Stigchel and Nijboer, 2017; Quinn et al., 2018; Morando et al., 2019; Spreij et al., 2020; Ten Brink et al., 2020; Villarreal et al., 2020). The CB methods were able to detect USN cases that the PnP did not, either due to compensatory strategies implemented by the patients or due to ceiling effects (Marshall et al., 1997; Deouell et al., 2005; Rengachary et al., 2009; Rabuffetti et al., 2012; Bonato et al., 2013; Van Kessel et al., 2013); especially in chronic (Andres et al., 2019) mild (Rengachary et al., 2009; Mizuno et al., 2016) and subclinical cases (Van Kessel et al., 2013; Machner et al., 2018).

### Specificity/reliability

Specificity was reported in 3/28 studies; however, only two studies included specificity percentage reports (high) (Quinn et al., 2018; Spreij et al., 2020) and only the latter included predictive values. Similarly, 2/28 studies reported high reliability; both of these studies report intra-rater reliability measurements (Jee et al., 2015; Morando et al., 2019), but only one also mentioned inter-rater reliability scores.

### Group differences

The ability of the CB task indices/variables to distinguish among patient groups (LBD+, LBD-, RBD+, RBD-) was explored by 22/28 studies, with 2/28 reporting limited (Liang et al., 2007; Chiba et al., 2010) and 20/28 stronger ability (Marshall et al., 1997; Rabuffetti et al., 2002, 2012; Deouell et al., 2005; List et al., 2008; Erez et al., 2009; Rengachary et al., 2009; Vossel et al., 2010; Bonato et al., 2013; Van Kessel et al., 2013; Pallavicini et al., 2015; Chung et al., 2016; Mizuno et al., 2016; Ten Brink et al., 2016, 2020; Stigchel and Nijboer, 2017; Machner et al., 2018; Andres et al., 2019; Spreij et al., 2020; Villarreal et al., 2020; Pierce et al., 2021). In a similar way, 18/28 studies discussed the ability of CB tasks to detect USN patients among control groups with the majority of the studies reporting positive results (Marshall et al., 1997; Rabuffetti et al., 2002, 2012; Deouell et al., 2005; List et al., 2008; Erez et al., 2009; Rengachary et al., 2009; Vossel et al., 2010; Ulm et al., 2013; Van Kessel et al., 2013; Vaes et al., 2015; Chung et al., 2016; Mizuno et al., 2016; Ten Brink et al., 2016; Machner et al., 2018; Spreij et al., 2020; Villarreal et al., 2020).

### Conventional or PnP comparison

Many studies compared CB tasks with the BIT (Deouell et al., 2005; Liang et al., 2007; Vossel et al., 2010; Van Kessel et al., 2013; Mizuno et al., 2016), with a combination of two or more original or similar subtests such as letter/star/line cancellation, line bisection, drawing and reading tasks (Marshall et al., 1997; Rabuffetti et al., 2002, 2012; Chiba et al., 2010; Ulm et al., 2013; Pallavicini et al., 2015; Morando et al., 2019). Some studies used only cancellation tasks (List et al., 2008; Bonato et al., 2013) or cancellation task(s) and line bisection (Quinn et al., 2018; Morando et al., 2019; Pierce et al., 2021). Other important tasks used as comparisons would be the baking tray and clock drawing task (Rengachary et al., 2009). Some studies compared CB tasks with both functional (e.g., CBS) (Erez et al., 2009; Ten Brink et al., 2016; Machner et al., 2018; Spreij et al., 2020) and PnP assessment (e.g., BIT) or with other widely used CB tools (e.g., TAP, CB, cancellation and line bisection) (Stigchel and Nijboer, 2017; Andres et al., 2019).

### Correlation coefficient

Correlation coefficients were reported by 15/28 studies using data from the CB tasks comparing them to conventional methods. Most of these report positive correlations (Rabuffetti et al., 2002; Liang et al., 2007; Erez et al., 2009; Van Kessel et al., 2013; Chung et al., 2016; Ten Brink et al., 2016, 2020; Machner et al., 2018; Morando et al., 2019; Spreij et al., 2020; Pierce et al., 2021), whereas in some cases there were more mixed results (List et al., 2008; Vossel et al., 2010; Stigchel and Nijboer, 2017). A variety of CB tasks (e.g., TOJ, conjunction/feature, Posner, etc.) were found to be highly correlated with cancellation tasks (Erez et al., 2009; Vossel et al., 2010; Stigchel and Nijboer, 2017; Machner et al., 2018; Ten Brink et al., 2020) and similarly with the CBS test (Erez et al., 2009; Machner et al., 2018; Spreij et al., 2020; Ten Brink et al., 2020).

### General benefits of CB assessment

The results of this investigation show that CB methods have been shown in many cases to be feasible (Rabuffetti et al., 2002; Deouell et al., 2005; Vossel et al., 2010; Jee et al., 2015; Chung et al., 2016; Morando et al., 2019), flexible (List et al., 2008; Vaes et al., 2015), valid (Erez et al., 2009; Ulm et al., 2013; Jee et al., 2015; Morando et al., 2019; Villarreal et al., 2020), reliable (Vossel et al., 2010; Jee et al., 2015; Morando et al., 2019) and user-friendly tools (List et al., 2008; Rabuffetti et al., 2012; Ulm et al., 2013; Pallavicini et al., 2015; Vaes et al., 2015).

This review confirms that CB assessment can provide important patient information and reveal more aspects of neglect symptoms than PnP assessment (Liang et al., 2007; Erez et al., 2009; Chung et al., 2016; Quinn et al., 2018), such as severity (Rengachary et al., 2009; Pierce et al., 2021), quantitative and qualitative data regarding patient behavior (Andres et al., 2019). Moreover, CB tasks can identify temporal, spatial, and non-spatial search strategy dynamics, helping to differentiate between different subtypes (Mizuno et al., 2016; Stigchel and Nijboer, 2017) with a relatively short administration time (Liang et al., 2007; List et al., 2008; Vossel et al., 2010; Ulm et al., 2013; Pierce et al., 2021).

### Limitations of the studies

The majority of the studies reviewed had a relatively small sample size (e.g., less than 50 participants) for demonstrating the clinical validity of the CB tests and control demographics among patients (List et al., 2008; Ulm et al., 2013; Jee et al., 2015; Vaes et al., 2015; Machner et al., 2018; Quinn et al., 2018). However some studies had smaller sample sizes (Rabuffetti et al., 2002; Deouell et al., 2005; Chiba et al., 2010; Bonato et al., 2013; Pallavicini et al., 2015; Mizuno et al., 2016; Andres et al., 2019; Morando et al., 2019; Pierce et al., 2021) and a number of authors did not include an unimpaired control group (Marshall et al., 1997; List et al., 2008; Chiba et al., 2010; Bonato et al., 2013; Pallavicini et al., 2015; Stigchel and Nijboer, 2017; Quinn et al., 2018; Andres et al., 2019; Morando et al., 2019; Ten Brink et al., 2020). In specific studies, no clear attempt was made to compare with a PnP task (Vaes et al., 2015; Ten Brink et al., 2016) or only one or two comparison tasks were conducted (Marshall et al., 1997; Jee et al., 2015; Vaes et al., 2015; Chung et al., 2016; Morando et al., 2019); comparing the CB with a battery of tasks would have increased sensitivity. Practical issues are among the most important drawbacks for example, CB tasks can be impractical to perform in a clinical setting, especially where these require large/expensive equipment rather than a typical computer and monitor (Ulm et al., 2013; Van Kessel et al., 2013; Jee et al., 2015; Vaes et al., 2015; Spreij et al., 2020). Selection bias is another potential concern in cases where samples do not represent the entire stroke population (e.g., acute/subacute/chronic or RBD/LBD) (Deouell et al., 2005; List et al., 2008; Erez et al., 2009; Vossel et al., 2010; Chung et al., 2016; Quinn et al., 2018). Other limitations include follow-up absence, covering only specific neglect component (Jee et al., 2015; Pallavicini et al., 2015; Vaes et al., 2015), not controlling demographics (Van Kessel et al., 2013; Jee et al., 2015; Mizuno et al., 2016) and factors such as hemianopia (Vossel et al., 2010; Stigchel and Nijboer, 2017; Spreij et al., 2020). Only 7/28 studies had a low risk of bias (Rengachary et al., 2009; Rabuffetti et al., 2012; Ten Brink et al., 2016, 2020; Machner et al., 2018; Villarreal et al., 2020; Pierce et al., 2021) the rest of them had moderate (Rabuffetti et al., 2002; Deouell et al., 2005; Liang et al., 2007; List et al., 2008; Erez et al., 2009; Vossel et al., 2010; Bonato et al., 2013; Ulm et al., 2013; Van Kessel et al., 2013; Chung et al., 2016; Stigchel and Nijboer, 2017; Quinn et al., 2018; Spreij et al., 2020) and unclear (Marshall et al., 1997; Chiba et al., 2010; Jee et al., 2015; Pallavicini et al., 2015; Vaes et al., 2015; Mizuno et al., 2016; Andres et al., 2019; Morando et al., 2019).

## Discussion

In this review, the main objective was to provide a summary and critical analysis of the existing evidence around CB assessment of USN, by investigating the shortcomings and strengths of the approaches followed by previous studies. Another purpose of our review was to generate fresh insight into our understanding of CB tasks and enhance future designs by demonstrating essential indications regarding clinical applicability and utility of CB assessment of USN.

One of our most important findings relates to the task type; most of the studies preferred to use CB versions of conventional tasks, however, it is revealed that CB tasks with more advanced designs such as RT and visual search tasks (e.g., feature and conjunction) can be more effective in detecting neglect symptoms. It was revealed that these types of tasks can capture more mild/chronic/subclinical cases than simple versions. Similarly, more complex designs, combining a variety of task types with different task demands, can maximize sensitivity by providing a wider data collection. These results are in line with existing evidence that presenting greater task difficulty can enhance sensitivity (Bonato, 2012; Buxbaum et al., 2012). These findings also support the work of other studies in the area demonstrating that divergent tasks can capture distinct components of this multifactorial syndrome (Sacher et al., 2004) and diversity of task demands can highlight disparate deficits associated with USN (Dukewich et al., 2012).

The results of this study explore the potential superiority of some outcome measures' sensitivity for detecting spatial bias and visual search deficit, such as RT and accuracy for visual search and comparable target detection tasks. This has previously been observed in a variety of other studies exploring the advantages of these measures (Bartolomeo et al., 1998; Anderson et al., 2000; Schendel and Robertson, 2002). Similarly, it was revealed in our review that in cancellation tasks and other CB versions of PnP, the CoC was among the most sensitive. These results are also in accordance with a wide background of evidence highlighting the benefits of capturing CoC in cancellation tasks (Rorden and Karnath, 2010; Suchan et al., 2012; Dalmaijer et al., 2015).

One of our objectives was to investigate features that optimize CB task design, but we were unable to demonstrate how some factors such as task duration, size of the display, and apparatus affect the effectiveness of these tasks. However, we concluded that most of the tasks last between 10 and 20 min, use a display with a 13–19 inches diagonal screen size, at a distance of 40–70 from the subject depending on the task design. Previous studies have reported that a short administration time can be more practical for a clinical environment and can avoid fatigue effect which can affect the patients' outcomes (Pedroli et al., 2015; Grattan and Woodbury, 2017). It was also observed that there are three main types of apparatus with the most used being a classic PC/laptop-monitor combination, the second most explored was the graphic tablet, and finally, the last decade has seen the introduction of touchscreen smartphone/tablet app tasks. It is important to consider various factors regarding hardware selection to enhance cost-effectiveness, feasibility and minimize practical issues affecting the clinician and patient (Tsirlin et al., 2009; Bonato and Deouell, 2013). The importance of minimizing motor and visuomotor task demands in order to avoid any contributing factors related to movement limitations is highlighted by the majority of the tests requiring a simple manual response either through a touchscreen, mouse, or response box/button. Previous research has established that increased motor demands can affect performance or cause motor bias to neglect patients with coexisting conditions such as directional hypokinesia (Mattingley et al., 1998; Sapir et al., 2007). One challenge influencing the optimization of CB tasks design is finding the right balance between the quantity and quality of data. For instance, a wider data collection would provide more information about the patient but could also increase the task duration, which could cause fatigue and affect the quality of the data.

As mentioned in the review most of the studies chose the CBS, cancellation, and line bisection tasks as a conventional comparison tool, these tests being among the most widely investigated existing neglect assessment tools with relatively high sensitivity scores (Bailey et al., 2000; Sarri et al., 2009; Chen et al., 2012; Azouvi, 2017). Several CB tasks were highly correlated with the CBS and cancellation tasks, and some studies reported varied results, which can be explained considering the lack of gold standard and the comparison with tasks requiring different demands and performance components. However, in order to accurately evaluate the diagnostic accuracy of a task, it is recommended to follow methodologies such as the ones summarized by Umemneku Chikere et al. (2019) in the absence of a golden standard. The CB tasks were overall more sensitive than conventional tools and could distinguish different patients groups (LBD, RBD+, RBD- etc.) from each other and from unimpaired control subjects, which corroborate the findings of a great deal of previous work (Schendel and Robertson, 2002; Dawson et al., 2008; Bonato, 2012; Bonato and Deouell, 2013). In the course of this review, we also discovered that CB tasks can collect a broad spectrum of data and provide more information about the patient's profile and behavior than PnP tasks. However, very little data were found in the literature around the specificity and reliability of CB tasks, and it was not possible to draw a conclusion regarding this. As presented in the review, CB tasks can provide greater flexibility than PnP (e.g., by projecting stimuli and testing neglect in near vs. far space). However, they operate in extra-personal space, in a similar way to PnP tasks, and cannot address all spatial representations relevant to the neglect syndrome (e.g., personal neglect).

The results presented here highlight the superiority of CB tasks compared to conventional methods in many domains, which is not unexpected considering most conventional tasks were designed and revised many years ago (e.g., the widely used BIT created by Wilson et al. in 1987). In recent years, the global technological expansion and digitalization of everyday life have minimized the practical constraints of CB assessment concerning requirements for hardware access. Additionally, technological advances have allowed the creation of more accessible designs, with some authors reducing further the practical boundaries by providing access to online/offline software for analysis and assessment (Rorden and Karnath, 2010; Dalmaijer et al., 2015). The reduction in barriers for the implementation of CB tasks and the evidence supporting their advantage in many domains, especially in detecting mild and chronic neglect cases, demonstrate that this method should be introduced to clinical settings. However, we do not suggest the total removal of conventional methods, since they can overcome practical restrictions (e.g., need for hardware) when CB methods are not necessary, such as in severe and acute cases. It can therefore be assumed that an optimized future model will include a combination of both conventional and CB assessment tools.

The evidence presented in this review demonstrates that CB neglect assessment methods are feasible, valid, flexible, reliable, and user-friendly tools. Our results are in accordance with the findings of multiple studies exploring the advantages of these methods (Halligan and Marshall, 1989; Kerkhoff and Marquardt, 1995; Donnelly et al., 1999; Guest et al., 2000; Smit et al., 2013; Van der Stoep et al., 2013; Hopfner et al., 2015).

### Limitations

Most of the included studies suffer similar limitations. In order to overcome these issues and minimize bias, it is recommended that future studies avoid selection bias by including a consecutive or random large sample of control subjects and patients from a wide stroke population with equivalent demographics (Chassé and Fergusson, 2019). It is also important to include more than two sensitive conventional tools as comparisons since there is no accepted gold standard. Follow-up testing in order to explore effectively clinical validity, psychometric properties, and accuracy of the tasks would also be welcome (Umemneku Chikere et al., 2019). The main weakness of our study is that we could not perform a meta-analysis due to the high heterogeneity among index/comparison tests, study data types, and methodologies of analysis. However, we performed a vote counting analysis based on a direction of effect plot, which can be used to synthesize evidence when there is a lack of data consistency across the selected studies; this method is considered appropriate though less powerful than methods that include *p-*values (McKenzie and Brennan, 2019). Even though the QUADAS-2 tool seemed like the optimal tool it was designed for diagnostic accuracy studies and the heterogeneity of the studies affected the risk of bias decision, since the authors could not answer the questions confidently. The reviewers did not expand the inclusion criteria to include VR assessment studies, which would have increased the quantity of data. However, this allowed a more distinctive focus to be applied to the review on CB assessment. Virtual reality-based assessment of USN has already been the focus of similar previous work (Tsirlin et al., 2009).

## Conclusion

Our review of major studies confirmed that CB assessment of USN can offer higher acceptability, flexibility, and feasibility compared to conventional methods. Extensive data show that CB methods have been proved to provide a wider variety of data than PnP tasks which can be crucial in understanding patients' profile and severity, as well as monitor progress and the effect of rehabilitation. There is a strong body of evidence demonstrating that CB assessment can be more sensitive and overcome conventional methods' practical issues such as compensatory strategies and ceiling effects, especially with more complex designs and combinations of different task types. The results obtained here may have implications for understanding essential features affecting the efficacy of CB tasks and indications can be implemented as a guide to improving future designs. The findings of this review complement those of earlier studies and suggest that CB tasks for USN assessment should be implemented in clinical settings.

## Data availability statement

The original contributions presented in the study are included in the article/Supplementary material, further inquiries can be directed to the corresponding author/s.

## Author contributions

IG and DP conceived the presented idea. IG developed the methodology, conducted a comprehensive literature search, performed and expressed the data synthesis of the selected studies, and wrote the original draft. IG and DL performed the final study selection, data extraction, and quality appraisal of the studies. DP verified the findings of this work and was involved in expert review and editing. All authors contributed to the article and approved the final manuscript.

## Funding

The authors received financial support for the publication of this article from the University of Birmingham.

## Conflict of interest

The authors declare that the research was conducted in the absence of any commercial or financial relationships that could be construed as a potential conflict of interest.

## Publisher's note

All claims expressed in this article are solely those of the authors and do not necessarily represent those of their affiliated organizations, or those of the publisher, the editors and the reviewers. Any product that may be evaluated in this article, or claim that may be made by its manufacturer, is not guaranteed or endorsed by the publisher.

## Abbreviations

BIT: behavioral inattention test
CB: computer-based
CBS: Catherine Bergego scale
CoC: center of cancellation
CVA: cerebrovascular accident
LBD: Left brain damaged
PnP: paper-and-pencil
PRISMA: preferred reporting items for systematic reviews and meta-Analysis
QUADAS-2: quality assessment tool for diagnostic accuracy studies-2
RBD: right brain damaged
RT: reaction time
TAP: test of attentional performance
TBI: traumatic brain injury
TIA: transient ischaemic attack
TOJ: temporal order judgment
USN: unilateral spatial neglect
–: without USN
+: with USN
VR: virtual reality.
