# Supplementary material for: Computer-based assessment of unilateral spatial neglect: A systematic review
Source: Front Neurosci. 2022 Aug 19;16:912626. doi: 10.3389/fnins.2022.912626 (PMC9437703; doi:10.3389/fnins.2022.912626)
Supplement: Supplementary file 1 [file Table_1.DOCX]

Supplementary Material

# Supplementary Tables

## Supplementary Table 1. Data extraction sheet.

| STUDY DETAILS |  |
| --- | --- |
| AUTHOR/YEAR |  |
| OBJECTIVES |  |
| PARTICIPANTS (CHARACTERISTICS/TOTAL NUMBER) |  |
| PATIENTS EXCLUDED |  |
| PATIENT DEMOGRAPHICS |  |
| SETTING |  |
| INDEX TEST |  |
| INDEX TEST TYPE |  |
| APPARATUS |  |
| TASK DETAILS |  |
| SESSIONS/DURATION |  |
| OUTCOME MEASURE |  |
| COMPARISON TESTS |  |
| DIAGNOSTIC ACCURACY MEASURES |  |
| PSYCHOMETRIC PROPERTIES |  |
| RESULTS |  |
| CONCLUSION |  |
| RISK OF BIAS |  |
| LIMITATION |  |
| NOTES |  |

Data Extraction Sheet Example.

| STUDY DETAILS | “Is the Posner Reaction Time Test More Accurate Than Clinical Tests in Detecting Left Neglect in Acute and Chronic Stroke?” |
| --- | --- |
| AUTHOR/YEAR | Jennifer Rengachary, MSOT, Giovanni d’Avossa, MD, Ayelet Sapir, PhD, Gordon L. Shulman, PhD, Maurizio Corbetta, MD. 2009 |
| OBJECTIVES | “Compare the diagnostic accuracy of common clinical tests for assessing left neglect compared to the computer-based reaction time Posner test to stroke survivors.” |
| PARTICIPANTS (CHARACTERISTICS/TOTAL NUMBER) | Patients with acute stroke (n=59) with left neglect  healthy age-matched controls (n=30). |
| PATIENTS EXCLUDED | – |
| PATIENT DEMOGRAPHICS | – |
| SETTING | – |
| INDEX TEST | Computerized reaction time tests. |
| INDEX TEST TYPE | Posner Cueing Paradigm. |
| APPARATUS | Computer. |
| TASK DETAILS | “The onset of a new trial was signaled by a color change, from red to green, of the fixation cross. Then 800 milliseconds later, an arrow cue pointing left or right appeared at fixation for 2360 milliseconds. After a delay ranging from 1000 to 2000 milli- seconds, the target (an asterisk) appeared for 300 milliseconds within 1 of the 2 frames (left or right). On 75% of the trials, the target appeared at the location indicated by the cue (valid condition), while on 25% of the trials, it appeared at the opposite location (invalid condition). Patients had to detect the target as quickly as possible with a right-hand keypress. The RTs were recorded. “ |
| SESSIONS/DURATION | A 1-hour testing session |
| OUTCOME MEASURE | RT, Accuracy score. |
| COMPARISON TESTS | Line cancellation, Behavioral Inattention Test. Mesulam Test, Clock drawing test, baking tray test, Shape test, Fluff test. |
| DIAGNOSTIC ACCURACY MEASURES | Sensitivity and Specificity |
| PSYCHOMETRIC PROPERTIES | – |
| RESULTS | “Most clinical tests were adequately accurate at the acute stage, but many were near chance at the chronic stage. The Posner test was the most sensitive test at both stages. The most sensitive variable was the reaction time difference for detecting targets appearing on the left compared with the right side.” |
| CONCLUSION | “Computerized reaction time tests can be used to screen for subtle but potentially clinically relevant left neglect, which may not be detectable by conventional clinical tests, especially at the chronic stage. Such tests may be useful to assess the severity of the patients’ deficits and provide more accurate measures of the degree of recovery in clinical trials than established clinical measures. “ |
| RISK OF BIAS | Low. |
| LIMITATION | – |
| NOTES | “This study found that a single, rapidly administered reaction time test showed the highest accuracy of all the tests we evaluated in classifying patients with neglect at both acute and chronic stages, and this accuracy was significantly better than that of many clinical tests at the chronic stage. “ |
