# Supplementary material for: Computer-based assessment of unilateral spatial neglect: A systematic review
Source: Front Neurosci. 2022 Aug 19;16:912626. doi: 10.3389/fnins.2022.912626 (PMC9437703; doi:10.3389/fnins.2022.912626)
Supplement: Supplementary file 2 [file Table_2.DOCX]

Supplementary Material

# Supplementary Tables

# 1.2. Supplementary Table 2. Summary of included studies Table Notes: BIT: Behavioural Inattention Test, BD: Brain damage, CB: Computer-based, CBS: Catherine Bergego Scale, CoC: Centre of cancellation, D(1,2,3,4): Domain (1,2,3,4), L: Left, R:Right., USN: Unilateral spatial neglect,), HR: Hit rate, NET: Neglect test, PnP: Paper-and-Pencil tasks, Pt: Patient/patients, RT: Reaction time, TAP: Test of Attentional Performance, -: without USN, +: with USN.

| TABLE 2. SUMMARY OF INCLUDED STUDIES. | | | | | |  |
| --- | --- | --- | --- | --- | --- | --- |
| *STUDY/TASK TYPE* | **RISK OF BIAS** | **POPULATION** | **CONVENTIONAL METHOD** | **COMPUTER-BASED METHOD** | **RESULTS** | **CONCLUSION** |
|  |  |  |  | **1.COMPUTERISED VERSIONS OF PnP TASKS** | |  |
| Rabuffetti et al. (2002)  *Touchscreen Cancellation* | D1: Low  D2: Unclear  D3: Low  D4: Low | 10 controls  15 stroke pt  (5 LBD-  5 RBD-  5RBD+) | Line & letter cancellation  Line bisection Sentence reading | Four visuospatial exploratory tasks, cancelation tests ('sparse letter', 'row shape’, 'row letter' and 'sparse shape' test) presented on a PC with a touch-screen monitor. Measuring the percentage of omitted targets, touched distractors/target perseveration, crossing etc. | RBD+ pt had significantly (p < 0.05) higher positive measures for neglect than other groups (especially controls) in four CB tasks with different modalities *Kruskall–Walls* one way analysis revealed (p = 0.007, p = 0.019, p = 0.020) and a close relationship was found between CB & PnP, *Spearman R coefficient* (0.862). | This tool was proved to be a useful, easy to administer and possibly more sensitive tool than PnP for assessing USN. |
| Liang et al. (2007)  *Graphic Tablet Battery* | D1: Low  D2: Low  D3: Low  D4: Unclear | Stroke pt:  33 USN+  110 USN- | BIT | 14 overlays (A4 oversized) on a Wacom graphic tablet. Tasks were performed with a cordless pen, divided in 4 sub-groups (2 cancellation, 4 figure copying, 3 figure completion L/R and 2 drawing from memory tasks). | Low error rate (varying 3.3%, 6.8%, 0%) can be achieved in distinguishing between USN+ and USN- in more than half time than the BIT.  Features were found to be sensitive and significantly correlated (p < 0.05) to the detection of USN symptoms, revealing more aspects than the BIT. | This approach can provide a quick, objective, and sensitive assessment tool. This method can reveal more aspects of neglect than BIT and offer a reduction of the overall assessment time. |
| Chiba et al. (2010)  *CB Line Bisection* | D1: Unclear  D2: Low  D3: Low  D4: Unclear | 10 USN+ pt | Line bisection  Line cancellation Drawing task | Verbal line bisection (VLB) and VLB & pointing task was performed on a 15.4-inch laptop computer (45cm distance). Participants were asked to define their subjective midpoint of a line, or a phrase and mean deviation was measured. | A *paired Student’s t-test* comparison revealed no significant difference in mean deviation in the two tasks as a group. Four pt had significantly (p < 0.05) greater rightward deviation in the VLB task plus pointing than the VLB and 2 in the opposite. | These tasks successfully assessed visual and proprioceptive bias, identifying perceptual-attentional bias of neglect. |
| *Rabuffetti et al. (2012)*  *Touchscreen Cancellation* | D1: Low  D2: Low  D3L Low  D4: Low | 119 controls  193pt  (72 LBD-  66 RBD-  55 RBD+) | Line & letter cancellation  Sentence reading  Line bisection | A computerized cancellation test on a pc with 19” touchscreen interface. Each participant performed randomly, two tests letter and shapes distribution. Number of touched targets/distractors, revisits and omissions were measured among other parameters. | An across-group *Kruskall-Walls* analysis of variance was found significant (p < 0.01) for all indexes except one. RBD+ compared to control and RBD- pt showed significantly more abnormal scores. Analysis revealed not significantly (p = 0.22; x2 test), but higher sensitivity of the CB than PnP. | The task can detect spatial exploration deficits with higher sensitivity than PnP, even in patients without USN in clinical testing. |
| *Ulm et al. (2013)*  *CB Battery* | D1: Low  D2: Low  D3: Low  D4: Unclear | 10 USN+ Stroke pt  10 controls | NET (german BIT):  Star cancellation  Line bisection  Figure copying  Clock drawing | Circle monitor was a 10–15 minutes assessment performed in 8 touchscreens in a circle. It included 4 different tests in the star cancellation test, line bisection, dice task (similar to baking tray), puzzle test (based on the hooper visual organization test). Each task captured among other measures omissions, average bisection position, deviation scores and correct selection respectively. | Both standard and CB version of the star cancellation test and line bisection differed reliably between patients and healthy controls (p = 0.025, p = 0.001), the dice task did not. Significant discriminant function was revealed for the CB (66.1% p = 0.003) and not significant (43,9% p > 0.05) for the NET (PnP). Most patients and controls rated the test as user-friendlier than PnP. | The test was proved to be a sensitive, well-accepted tool capturing quickly and accurately visual neglect symptoms with greater diagnostic validity than PnP. |
| *Jee et al. (2015)*  *CB Line Bisection* | D1: Unclear  D2: Low  D3: High  D4: Unclear | 30 controls  11 RBD+ | Line bisection | Line bisection test in a semi-computerized e-system performed though a pc, an electronic pen and micro pattern printed paper. Capturing the percent of deviation (%) and assessment duration. | High levels of reliability were found. Inter-rater (nearly perfect) observer agreement between the e-system and raters for all tests, varied from 0. 84 to 0.98 (p < 0.001) and the intra-rater (test-retest) reliability from 0.84 to 0.91 (p < 0.001) for the patient group. | It was found to be a valid and reliable assessment method that can feasibly replace traditional PnP. |
| *Pallavicini et al. (2015)*  *Touchscreen App Battery* | D1: Low  D2: Unclear  D3: Low  D4: Unclear | 8 RBD+  8 RBD- | Line & star cancellation  Conventional card dealing task | ‘Neglect App’ task performed on an iPad2 touchscreen with stylus. Including a simple line cancellation and cancellation with distractors test (similar to star cancellation) and a CB card dealing task. Measuring omissions and number of correctly given cards respectively. | *Mann-Whitney U* test revealed that: The app versions of cancellation tests were similarly effective as PnP in neglect diagnosis both distinguishing significantly (p = 0.008) and (p = 0.001) respectively between groups. Similarly, the CB Card Dealing task (p < 0.05), but not the conventional (p = 0.755). | This app was found to be a sensitive and user-friendly tool for the detection of neglect symptoms. |
| *Vaes et al. (2015)*  *CB Battery* | D1: Unclear  D2: Low  D3: Low  D4 Unclear | 20 controls  20 stroke pt | Star cancellation  Line bisection | A digital USN test battery with prism measurements using a variety of equipment such as PC/laptop, response box and graphic tablet. It includes 9 tasks approximately 5min each (2 cancellation, 2 bisection tests, drawing, extinction, spatial memory, and a visuospatial navigation tests). Each captures a variety of measures such as CoC, deviation, correct responses, and RT. | Mann-Whitney U test revealed that 21 of the 26 test indexes/variables could significantly (p < 0.05) differentiate between neglect and control group, most of which could not be implemented in PnP tests. | This test offers user-friendly, flexible, standardized online measurements and performance monitoring of USN. Providing a wider collection of quantitative and qualitative data than PnP. |
| *Chung et al. (2016)*  *Touchscreen App* | D1: Low  D2: Low  D3: Low  D4: Unclear | 40 stroke pt  (20 RBD+  10 RBD-  10 LBD-)  10 controls | Line bisection  Star cancellation | The computerized table setting test (CTST) is performed in an iPad touchscreen. Subjects are required to set a table by dragging 12 dishes located below the table on the tablet screen (similar to the Baking Tray task). It captures measures like midline deviation, time, selection tendency. | Horizontal deviation was significantly higher for RBD+ compared to others. All 3 indexes of the CTST were significantly correlated with the total neglect score of conventional tasks (p < 0.01). Overall sensitivity of the CTST was 95% of Line Bisection test was 75% and of Star Cancellation test was 94.4%. | The test can be feasibly administered and is comparable with traditional test with higher sensitivity providing useful information for the diagnosis of neglect. |
| *Ten Brink et al. (2016)*  *CB Battery (Line Bisection & Cancellation)* | D1: Low  D2: Low  D3: Low  D4: Low | 37 controls  280 stroke pt  (115 LBD-  108 RBD−  18 LBD+  39 RBD+) | Digital:  Shape cancellation  Line bisection | Computerized shape cancellation test and line bisection test. Capturing omissions score, revisits, intersections, search direction, best r and average deviation. | Performance comparison revealed higher numbers of intersections for RBD+ than LBD+ (p = 0.009) and RBD- (p = 0.001) pt. Significantly higher numbers of intersections (p < 0.003) and omissions (p < 0.001) for RBD+ and LBD+ pt compared to controls. | Disorganized search was strongly related to USN, and the number of intersections was found to be the most sensitive measure for visual search deficits in stroke survivors. |
| *Quinn et al. (2018)*  *Touchscreen App (Line Bisection & Cancellation)* | D1: Low  D2: Low  D3: Low  D4: Unclear | 48 stroke pt | Albert’s test  Star cancelation  Line Bisection | The stroke vision app (Tarbert et al., 2014) is including tests for visual acuity, visual field and digital tests of visual inattention (line bisection and face cancelation) performed at a Google Nexus 10 tablet device (33cm distance). | Comparison of app-based method with PnP revealed: Similar acceptability scores. Moderate to good accuracy measures. Overall, 79% sensitivity and 88% specificity for detecting stroke visual field deficits. | The app was similarly good as the conventional methods and can be used as post-stroke visual impairment screening tool providing more information than PnP. |
| *Morando et al. (2019)*  Touchscreen App (Line Bisection & Cancellation*)* | D1: Unclear  D2: High  D3: Low  D4: Unclear | 10 pt | Line bisection  Albert’s test | Digital line bisection and an albert (Cancellation) Test through the remote monitoring validation engineering system (ReMoVES) platform on a touchscreen smartphone/tablet display. | Statistical analysis reported high test-retest reliability (higher than 0.75) for conventional PnP versions and similarly high Pearson Correlation Coefficient for digital versions line bisection (offset: = 0.83, Rate: p = 0.94), Albert’s test (uncrossed L/R: p = 0.98 / p = 0.80). | The severity of USN can be effectively monitored remotely for de-hospitalized patients by the novel tool. |
|  |  |  |  | **2.VISUAL SEARCH TASKS** |  |  |
|  |  |  |  | **2A. DUAL & DYNAMIC TASKS** |  |  |
| *Marshall et al. (1997)*  *Dual Dynamic Visual Search Task* | D1: Low  D2: Unclear  D3: Low  D4: Unclear | 36 stroke pt  (20 RBD  16 LBD)  20 controls | Clock drawing  Letter cancellation | *A 3 min* visual tracking task (keeping a blue cross in the centre of a white square, capturing accuracy), a 6min target detection task (identifying all yellow circles, measuring detected targets), a 6min dual task (both tasks), displayed on a computer monitor (21’’, 50-60 cm distance) with mouse. | RBD pt had worse performance (p = 0.03) than the other two groups in visual tracking task. The control group had significantly better performance than the pt groups on the single CB task (p < 0.004) and on the dual task (p < 0.02). | Conventional methods were found insensitive for detecting inattention. The dynamic task could identify impaired attention in a more sensitive way. |
| *Deouell et al. (2005)*  *RT Dynamic Visual Search Task* | D1: Low  D2: Low  D3: Low  D4: Unclear | 9 controls  32 RBD  16 LBD | BIT | The “Starry Night Test” (SNT) was performed in a 15” monitor (100cm distance, around 30min duration), through a response box. Patients had to detect dynamically appearing targets between blinking distractors and RT was captured. | 70% of the RBD pt had significantly prolonged left RT (p < 0.05) and as a group performed significantly worse than the LBD pt and control group (p < 0.001). In 50% of the cases SNT showed significant side differences and the BIT did not. | The test was proved to be feasible for hospitalized patients and more sensitive than conventional tests, providing quantitative data of neglect severity and recovery. |
| *Bonato et al. (2013)*  *Dual Task* | D1: Unclear  D2: Low  D3: Low  D4: Low | 10 stroke RBD | BIT (3 cancellation subtests) | The CB paradigm captured the percentage of correct detections for:  single task (verbal report of the target position). Visual dual task (letter identification before reporting target position). Auditory dual task (count an announced number by 2 twice before reporting target position). | A significant L/R difference between target detection is reported (p < 0.001). Percentage of correct left target was significantly decreased in dual tasks (p < 0.05). Cancellation tasks revealed less overall omissions to the left (6.9%) than the CB task (60.2%). | CB attention-demanding tasks can avoid compensatory strategies implemented by stroke survivors and have higher sensitivity than conventional cancellation tasks. |
| *Van Kessel et al. (2013)*  *RT Dual & Driving Simulator Task* | D1: Unclear  D2: Low  D3: Low  D4: Low | 43 stroke pt  (22 LBD  21 RBD)  20 Controls | BIT | Computerized visual reaction time single (CVRT) and dual (CVRT-D) task (van Kessel et al., 2010) was performed with the use of a steering wheel, projector, a screen, and a pc. It was a 5 min dynamic lane tracking (CVRT) and a continuous RT task where patients had to detect a rectangle (by pressing a button) while performing a driving simulator test (CVRT-D). Number of omissions and RT asymmetries, ipsilesional RT were measured. | Significant Pearson Correlation between BIT and contralesional omissions were revealed CVRT (p < 0.05) and CVRT-D (p < 0.005).  Significant Pearson Correlation in pt groups and RT asymmetries were detected in the single (p < 0.01) and dual task (p < 0.05).  The CVRT-D task was able to detect cases with no symptoms on the BIT and CVRT. | CVRT test was sensitive in discriminating RBD pt group and was able to overcome the compensational strategies. CVRT-D can detect also mild cases of neglect without clinical signs. The RT asymmetry scores were found to be sensitive for spatial bias detection. |
| *Andres et al. (2019)*  *Dual Task* | D1: High  D2: Unclear  D3: Low  D4: Low | 2 RBD- pt  6 Controls | Bells test,  TAP (visual neglect)  Apple test  Line bisection | Dual task (Bonato et al., 2010; Bonato et al., 2013) performed in a Dell laptop (17’ screen, 60 cm distance) with Psychopy software. | Normal range patient performance was found at PnP and simple CB task (TAP). Although single task omissions were not significant (p = 0.15), the dual-task revealed significantly (p < 0.001) the neglect/extinction symptoms for 2 cases. | Dual tasks have higher sensitivity than PnP and simple CB tasks, capturing more quantitative and qualitative data of spatial attention deficits especially in chronic stages. |
| *Villarreal et al. (2020)*  *Dual Dynamic Task* | D1: Low  D2: Low  D3: Low  D4: Low | 40 USN+ pt  (20 LBD  20 RBD)  20 controls | Bells test | The 2 min detection task (reacting to a red sphere flashing among others appearing and to a number) and 4 min crash task (reacting to a collision of two moving grey spheres resulting in a white flash and to number sequence). The tasks were presented on a video projector capturing HR, correct reactions, RT and omissions. | No significant group differences in bells test performance were found. RBD pt had significantly increased left hemispace HR than controls in both crash (p = 0.12) and detection (p = 0.033) tasks. All groups had significantly (p < 0.001) slower RT in complex tasks. | Dual tasks were found to have higher sensitivity than conventional tasks, revealing subclinical neglect in stroke. |
|  |  |  |  | **2B. FEATURE & CONJUCTION TASKS** | |  |
| *List et al. (2008)*  *Feature & Conjunction, RT, Visual Search Task* | D1: Low  D2: Low  D3: Low  D4: Unclear | 12 controls  23 unilateral BD pt | Line cancellation  Letter search  Symbol search | A 5-20 min feature search (detecting a blue circle among red distractors), a scattered feature search (detecting a red square among blue) and a conjunction search task (detecting a red square among red triangles and blue squares) on a laptop widescreen (60-70 cm distance). L/R Accuracy and RT were measured. | Healthy individuals demonstrated symmetricity of the test and normative data were collected. Feature search task (p = 0.07) was not significant in detecting contralesional deficits in patients, but in the Conjunction task RBD pt performed significantly worse than the LBD pt (p < 0.05) and controls. A variety of test performance was detected across sessions. | The adaptive procedure successfully detected lateralized visual deficits (even subtle) and had relatively quick administration time. The Conjunction task was found to be more sensitive in detecting lateralized deficits than the feature task. |
| *Erez et al. (2009)*  *Feature & Conjunction RT, Visual Search Task* | D1: Low  D2: Low  D3: Low  D4: Unclear | 72 Stroke pt  (25 RBD+  27 RBD-  20 LBD-)  39 controls | BIT  CBS | Visual spatial search task (VISSTA) includes a feature (detecting a red circle among blue) and a conjunction task (detecting a red circle among blue circles and red squares). The apparatus included a computer screen and a response button, capturing HR and RT. | RBD+ pt showed a significant (p < 0.001) contralesional disadvantage in feature and conjunction HR and RT than the other groups. All groups had worse mean RT in the conjunction than the feature task (p < 0.001) Pearson Correlation coefficients of HR and cancellation task were significant for feature task in RBD pt and for conjunction in all groups and similarly only in RBD+ pt for CBS. | Combining CB assessment with PnP can increase sensitivity and provide useful information for visual-spatial inattention. |
|  |  |  |  | **2C. STATIC VISUAL SEARCH** |  |  |
| *Mizuno et al. (2016)*  *CB Cancellation and RT Visual Search* | D1: Low  D2: Unclear  D3: Low  D4: Unclear | 2 USN+  1 USN-  16 Controls | BIT | Four tasks of visual exploration (2 cancellation, a visuomotor & visual search task). Participants were asked to detect targets on a 32-inch touchscreen display (45 cm distance) or by pressing a response button. Capturing measures such as RT, order of cancellation, omission, total cancellation time and CoC. | The CB test detected one viewer and one stimulus centred USN+ pt. Spatial and temporal patterns of cancellation between the two subtypes were compared revealing that the first had not significant rightward attention bias (longer left RT) (p > 0.05) while the second did (p < 0.01) in both visuomotor and visual search tests. | The CB test can provide information about USN patients’ temporal and spatial dynamics of visual search strategy, and can be more sensitive than PnP, detecting also mild USN cases. |
| *Machner et al. (2018)*  *Static Visual Search & Posner task* | D1: Low  D2: Low  D3: Low  D4: Low | 34 RBD pt  (10 USN-  12 moderate  12 severe neglect)  11 controls | French CBS  Line bisection  Text reading  Star & bell cancellation  Figure copying | The desk task is a naturalistic visual search task performed in 24-inch widescreen monitor. Participants are asked to detect a paperclip among 30 objects in a desk picture and respond by pressing a button (max 12s /100 desk images) measuring false alarms, detection rate etc. Subjects also performed a variant of RT Posner task. | The severe and the moderate neglect group showed significantly (p < 0.01) increasing durations and decreasing detection rate (p < 0.001, p < 0.05) from outmost right to outmost left. High correlation were found between Posner task, CBS and desk task, with mean search time for desk task (p < 0.001), latency index of Posner task (p < 0.001) and CoC of bell’s task (p < 0.001). | The desk task was found to be more sensitive than PnP by detecting also neglect patients without clinical signs. |
| *Ten Brink et al. (2020)*  *CB Static Visual Search Task* | D1: Low  D2: Low  D3: Low  D4: Low | 23 RBD +  55 RBD -  49 LBD - | Shape cancellation  Line bisection  CBS | In the visual search task participants had to find targets among distractors (performed on a 13.3-inch touchscreen laptop). Each trial varied in number of distractors and target location. The effect of these variants was evaluated through HR. | Increasing target number revealed significantly lower HR for RBD+ pt (p = 0.006). No effect of number of targets on HR was seen in patients with LBD pt (p = 0.063) and RBD- pt (p = 0.015). Moderate positive relation was found between the CB task and PnP task (p < 0.001) and CBS (p < 0.001). | Additional targets reduce HR and can increase the sensitivity of USN visual search tasks. |
|  |  |  |  | **3.DIFFERENT TYPES OF TASKS** |  |  |
| *Rengachary et al. (2009)*    *Posner Cue Paradigm* | D1: Low  D2: Low`  D3: Low  D4: Low | 59 LUSN+ stroke pt 30 controls | Line & shape cancellation  Mesulam test  Clock drawing  Baking tray  Fluff test | Posner cueing paradigm (40 trials around 15 min) was performed in a computer with a 17-inch monitor through a response button box. Patients had to detect a target (left or right) in two square frames as quickly as possible. Accuracy and RT was measured. | The Posner task (L/R RT) was significantly (p < 0.05) more sensitive than most of the conventional methods in the acute stage and chronic stage, except Mesulam cancellation tasks which revealed similar sensitivity scores. | CB RT tasks can accurately detect and assess the severity of neglect even on mild and chronic cases that PnP could not. |
| *Vossel et al. (2010)*  *Extinction/ Neglect Task* | D1: Low  D2: Low  D3: Low  D4: Unclear | 56 RBD Stroke pt  18 controls | BIT  Confrontation technique | The neglect and extinction CB test was performed through a PC, a monitor, and a response button. Subjects had to respond whenever they detected a target (square) appearing unilaterally or bilaterally with a distractor (circle). The task duration was around 11,3 min and measured detection probabilities. | Controls had almost no errors. It was revealed that 23,2% of the pt had extinction, 17,86% moderate and 14,28% severe neglect. Low correlation between CB and confrontation technique, but high Pearson correlation between line bisection and cancellation tasks (p < 0.001) was detected. | The computerized test can be easily applied in a clinical setting and repeated reliably on separate occasions, identifying visual neglect and extinction. |
| Stigchel and Nijboer (2017)  *Temporal Order Judgment Task* | D1: Unclear  D2: Low  D3: Low  D4: Low | 73 stroke pt | Digital shape cancellation  Line bisection | In the temporal order judgment (TOJ) subjects had to detect which colour of a square (red or green) appeared first in a monitor display (90cm distance) by pressing two different response buttons. | Performance on TOJ revealed strong correlation (p < 0.01) with the shape cancellation (both can sensitively capture spatial bias), but not with the line bisection test (p = 0.08), which captures object-based deficits. TOJ minimized chances of ceiling flour increasing sensitivity. | The TOJ test can be used supplementary with the line bisection and shape cancellation test and capture spatial and non-spatial components of neglect in a more sensitive manner than PnP. |
| *Spreij et al. (2020)*  *Driving Simulator*  *Task* | D1: Low  D2: Unclear  D3: Low  D4: Low | 33 LUSN+  7 RUSN+  7  LRUSN+(Recovered)  53 USN-  21 controls | Shape cancellation CBS | The 2min steering wheel simulated driving task (Adapted from the computerized visual reaction time task by van Kessel et al., 2010) was projected on a large screen. Measures such as average road position and deviation were captured. Also omissions and asymmetry score between sides were captured by a digitised shape cancellation task of 54 targets and 75 distractors in different sizes and shapes. | The sensitivity was 51.5% for LUSN+ pt, but only 28.6 for RUSN+ pt. Specificity: 94.3% of USN- had normal performance, Positive predictive values for LUSN+ and RUSN+ pt were 85% and 40% respectively and negative were 75.8% and 90.9% respectively. Correlation between average position and neglect severity according to CBS was analysed (p = 0.05). | It is proposed to include several types of tasks rather in isolation. Dynamic tasks can effectively assess mild and chronic USN cases. |
| *Pierce et al. (2021)*  *Manual Exploration Touchscreen App* | D1: Low  D2: Low  D3: Low  D4: Low | 39 pt  (12 RBD+  17 LBD-  10 RBD-)  14 Controls | Bells & apple cancellation  Line bisection | The manual exploration task was performed on a touchscreen tablet. Participants had to tap on the screen using their index finger with their eyes closed until they found the target (a rectangular area) appearing in a random location (3 min). Measures such as percentage of time spent L/R, percentage of taps within five columns, and mean horizontal position. | No significant correlation was revealed between the manual exploration task and the line Bisection, bells, or apples cancellation. RBD+ pt (3/4) showed a significant rightward bias in CB task as compared to the other groups. USN+ pt (4/12) showed neglect signs in the CB tasks. Patients with severest neglect had poor performance on multiple tasks. | This is an easy and quick task to administer without visual input and can be used supplementary to conventional methods to assess the severity of neglect. |

Andres, M., Geers, L., Marnette, S., Coyette, F., Bonato, M., Priftis, K., et al. (2019). Increased Cognitive Load Reveals Unilateral Neglect and Altitudinal Extinction in Chronic Stroke. *Journal of the International Neuropsychological Society* 25(6)**,** 644-653. doi: 10.1017/S1355617719000249.

Bonato, M., Priftis, K., Marenzi, R., Umiltà, C., and Zorzi, M. (2010). Increased attentional demands impair contralesional space awareness following stroke. *Neuropsychologia* 48(13)**,** 3934-3940. doi: https://doi.org/10.1016/j.neuropsychologia.2010.08.022.

Bonato, M., Priftis, K., Umiltà, C., and Zorzi, M. (2013). Computer-Based Attention-Demanding Testing Unveils Severe Neglect in Apparently Intact Patients. *Behavioural neurology***,** 179-181. doi: 10.3233/BEN-2012-129005.

Chiba, Y., Nishihara, K., and Haga, N. (2010). Evaluating visual bias and effect of proprioceptive feedback in unilateral neglect. *Journal of Clinical Neuroscience* 17(9)**,** 1148-1152. doi: 10.1016/j.jocn.2010.02.017.

Chung, S., Park, E., Ye, B.S., Lee, H., Chang, H.-J., Song, D., et al. (2016). The Computerized Table Setting Test for Detecting Unilateral Neglect. *PloS one* 11**,** e0147030. doi: 10.1371/journal.pone.0147030.

Deouell, L.Y., Sacher, Y., and Soroker, N. (2005). Assessment of spatial attention after brain damage with a dynamic reaction time test. *Journal of the International Neuropsychological Society* 11(6)**,** 697-707. doi: 10.1017/S1355617705050824.

Erez, A., Katz, N., Ring, H., and Soroker, N. (2009). Assessment of spatial neglect using computerised feature and conjunction visual search tasks. *Neuropsychol Rehabil* 19(5)**,** 677-695. doi: 10.1080/09602010802711160.

Jee, H., Kim, J., Kim, C., Kim, T., and Park, J. (2015). Feasibility of a Semi-computerized Line Bisection Test for Unilateral Visual Neglect Assessment. *Applied Clinical Informatics* 6(2)**,** 400-417. doi: 10.4338/aci-2015-01-ra-0002.

Liang, Y., Guest, R.M., Fairhurst, M.C., and Potter, J.M. (2007). Feature-based assessment of visuo-spatial neglect patients using hand-drawing tasks. *Pattern Analysis and Applications* 10(4)**,** 361-374. doi: 10.1007/s10044-007-0074-x.

List, A., Brooks, J.L., Esterman, M., Flevaris, A.V., Landau, A.N., Bowman, G., et al. (2008). Visual hemispatial neglect, re-assessed. *Journal of the International Neuropsychological Society* 14(2)**,** 243-256. doi: 10.1017/S1355617708080284.

Machner, B., Koenemund, I., von der Gablent, J., Bays, P.M., and Sprenger, A. (2018). The Ipsilesional Attention Bias in Right-Hemisphere Stroke Patients as Revealed by a Realistic Visual Search Task: Neuroanatomical Correlates and Functional Relevance. *Neuropsychology* 32(7)**,** 850-865. doi: 10.1037/neu0000493.

Marshall, S.C., Grinnell, D., Heisel, B., Newall, A., and Hunt, L. (1997). Attentional deficits in stroke patients: A visual dual task experiment. *Archives of Physical Medicine and Rehabilitation* 78(1)**,** 7-12. doi: 10.1016/S0003-9993(97)90002-2.

Mizuno, K., Kato, K., Tsuji, T., Shindo, K., Kobayashi, Y., and Liu, M. (2016). Spatial and temporal dynamics of visual search tasks distinguish subtypes of unilateral spatial neglect: Comparison of two cases with viewer-centered and stimulus-centered neglect. *Neuropsychological Rehabilitation* 26(4)**,** 610-634. doi: 10.1080/09602011.2015.1051547.

Morando, M., Bonotti, E., Giannarelli, G., Olivieri, S., Dellepiane, S., and Cecchi, F. (2019). "Monitoring Home-Based Activity of Stroke Patients: A Digital Solution for Visuo-Spatial Neglect Evaluation: Proceedings of the 4th International Conference on NeuroRehabilitation (ICNR2018), October 16-20, 2018, Pisa, Italy."), 696-701.

Pallavicini, F., Pedroli, E., Serino, S., Dell'Isola, A., Cipresso, P., Cisari, C., et al. (2015). Assessing Unilateral Spatial Neglect using advanced technologies: The potentiality of mobile virtual reality. *Technology and Health Care* 23(6)**,** 795-807. doi: 10.3233/THC-151039.

Pierce, J.E., Ronchi, R., Thomasson, M., Rossi, I., Casati, C., Saj, A., et al. (2021). A novel computerized assessment of manual spatial exploration in unilateral spatial neglect. *Neuropsychological Rehabilitation*. doi: 10.1080/09602011.2021.1875850.

Quinn, T.J., Livingstone, I., Weir, A., Shaw, R., Breckenridge, A., McAlpine, C., et al. (2018). Accuracy and Feasibility of an Android-Based Digital Assessment Tool for Post Stroke Visual Disorders-The StrokeVision App. *Front Neurol* 9**,** 146. doi: 10.3389/fneur.2018.00146.

Rabuffetti, M., Farina, E., Alberoni, M., Pellegatta, D., Appollonio, I., Affanni, P., et al. (2012). Spatio-temporal features of visual exploration in unilaterally brain-damaged subjects with or without neglect: Results from a touchscreen test. *PLoS ONE* 7(2). doi: 10.1371/journal.pone.0031511.

Rabuffetti, M., Ferrarin, M., Spadone, R., Pellegatta, D., Gentileschi, V., Vallar, G., et al. (2002). Touch-screen system for assessing visuo-motor exploratory skills in neuropsychological disorders of spatial cognition. *Medical and Biological Engineering and Computing* 40(6)**,** 675-686. doi: 10.1007/BF02345306.

Rengachary, J., d'Avossa, G., Sapir, A., Shulman, G.L., and Corbetta, M. (2009). Is the posner reaction time test more accurate than clinical tests in detecting left neglect in acute and chronic stroke? *Arch Phys Med Rehabil* 90(12)**,** 2081-2088. doi: 10.1016/j.apmr.2009.07.014.

Spreij, L.A., Ten Brink, A.F., Visser-Meily, J.M.A., and Nijboer, T.C.W. (2020). Simulated driving: The added value of dynamic testing in the assessment of visuo-spatial neglect after stroke. *Journal of Neuropsychology* 14(1)**,** 28-45. doi: 10.1111/jnp.12172.

Stigchel, S., and Nijboer, T. (2017). Temporal order judgements as a sensitive measure of the spatial bias in patients with visuospatial neglect. *Journal of neuropsychology* 12. doi: 10.1111/jnp.12118.

Tarbert, C.M., Livingstone, I.A., and Weir, A.J. (2014). Assessment of visual impairment in stroke survivors. *Annu Int Conf IEEE Eng Med Biol Soc* 2014**,** 2185-2188. doi: 10.1109/embc.2014.6944051.

Ten Brink, A.F., Elshout, J., Nijboer, T.C., and Van der Stigchel, S. (2020). How does the number of targets affect visual search performance in visuospatial neglect? *Journal of Clinical and Experimental Neuropsychology* 42(10)**,** 1010-1027.

Ten Brink, A.F., van der Stigchel, S., Visser-Meily, J.M.A., and Nijboer, T.C.W. (2016). You never know where you are going until you know where you have been: Disorganized search after stroke. *Journal of Neuropsychology* 10(2)**,** 256-275. doi: 10.1111/jnp.12068.

Ulm, L., Wohlrapp, D., Meinzer, M., Steinicke, R., Schatz, A., Denzler, P., et al. (2013). A Circle-Monitor for Computerised Assessment of Visual Neglect in Peripersonal Space. *PLOS ONE* 8(12)**,** e82892. doi: 10.1371/journal.pone.0082892.

Vaes, N., Lafosse, C., Nys, G., Schevernels, H., Dereymaeker, L., Oostra, K., et al. (2015). Capturing peripersonal spatial neglect: An electronic method to quantify visuospatial processes. *Behavior Research Methods* 47(1)**,** 27-44.

Van der Stoep, N., Visser-Meily, J.M., Kappelle, L.J., de Kort, P.L., Huisman, K.D., Eijsackers, A.L., et al. (2013). Exploring near and far regions of space: distance-specific visuospatial neglect after stroke. *J Clin Exp Neuropsychol* 35(8)**,** 799-811. doi: 10.1080/13803395.2013.824555.

van Kessel, M.E., van Nes, I.J., Brouwer, W.H., Geurts, A.C., and Fasotti, L. (2010). Visuospatial asymmetry and non-spatial attention in subacute stroke patients with and without neglect. *Cortex* 46(5)**,** 602-612. doi: 10.1016/j.cortex.2009.06.004.

Van Kessel, M.E., Van Nes, I.J.W., Geurts, A.C.H., Brouwer, W.H., and Fasotti, L. (2013). Visuospatial asymmetry in dual-task performance after subacute stroke. *Journal of Neuropsychology* 7(1)**,** 72-90. doi: 10.1111/j.1748-6653.2012.02036.x.

Villarreal, S., Linnavuo, M., Sepponen, R., Vuori, O., Jokinen, H., and Hietanen, M. (2020). Dual-Task in Large Perceptual Space Reveals Subclinical Hemispatial Neglect. *Journal of the International Neuropsychological Society* 26(10)**,** 993-1005. doi: 10.1017/S1355617720000508.

Vossel, S., Eschenbeck, P., Weiss, P., and Fink, G. (2010). Assessing visual extinction in right-hemisphere stroke patients with and without neglect. *Klinische Neurophysiologie. Conference* 41(1).
