# Supplementary material for: Computer-based assessment of unilateral spatial neglect: A systematic review
Source: Front Neurosci. 2022 Aug 19;16:912626. doi: 10.3389/fnins.2022.912626 (PMC9437703; doi:10.3389/fnins.2022.912626)
Supplement: Supplementary file 3 [file Table_3.DOCX]

Supplementary Material

# Supplementary Tables

## 1.3 Supplementary Table 3. Summary Of main results: Table Notes: BIT: Behavioural inattention test, BD: Brain damage, CB: Computer-based, CBS: Catherine Bergego scale, CoC: Centre of cancellation, L: Left, R: Right, USN: Unilateral spatial neglect, HR: Hit rate, PnP: Paper-and-Pencil tasks, PSS: Point of subjective simultaneity, Pt: Patient/patients, RT: Reaction Time, -: without USN, +: with USN.

| **Authors/Year** | Sensitivity | Specificity | Reliability | Correlation Coefficients | Between groups differences. Sensitive Variables/ Features | Other reports: |
| --- | --- | --- | --- | --- | --- | --- |
| Marshall et al. (1997) |  |  |  |  | The visual tracking task can distinguish between RBD pt and other groups (p = 0.03), between pt and the control group (p < 0.02) It demonstrated significantly better control performance on the target detection single task (p < 0.004) and dual task (p < 0.02). |  |
| Rabuffetti et al. (2002) |  |  |  | Close relationship (Spearman R: 0.862) between PnP Cancellation test. | RBD+ pt had significantly higher neglect indexes (Crossing indexes, Latency indexes, Latency gradients significant group differences) than other groups (especially control) (p < 0.05). | Feasible |
| Deouell et al. (2005) | More sensitive than BIT (6/12 pt showed normal scores in the BIT and significant (p < 0.05) side difference). |  |  |  | RT can distinguish between RBD and LBD pt and the control group (p < 0.001). Significantly longer L RT for the RBD pt (p < 0.05).  Only the RBD pt group had L/R RT side difference (p < 0.001). |  |
| Liang et al. (2007) |  |  |  | More than 8 features were significantly correlated (p < 0.05) with neglect symptoms. | Error rate: Varying from (3.3%, 6.8% and 0%) |  |
| List et al. (2008) | The conjunction task was more sensitive than the feature task. |  |  | Correlation between PnP and conjunction search score was significant (p < 0.05) but not with the feature task. | Conjunction tasks can distinguish RBD from LBD pt (p < 0.05) and control group (p < 0.005) in contralesional deficits and search score. | Flexible, user-friendly, and quick. |
| Erez et al. (2009) | Conjunction tasks can be more sensitive than feature and conventional methods. |  |  | Pearson correlation coefficients were significant between feature/conjunction tasks (HR) and cancellation task performance, in RBD+ (p < 0.001) / p < 0.005) and RBD- pt (p < 0.001) / (p < 0.001). Similarly, between the CBS and feature/conjunction tasks (HR) (p < 0.005/ p < 0.001). | CB tasks can significantly distinguish RBD+ from RBD- pt, LBD pt and the control group in left RT and HR (p < 0.001) | Valid for spatial attention post-stroke. |
| Rengachary et al. (2009) | High accuracy (sensitivity/specificity). The Posner task was significantly more sensitive (p < 0.05) than most PnP in detecting chronic and acute neglect cases. | High specificity. |  |  | Posner L/R RT differences and average RT can significantly (p < 0.05) distinguish acute pt (AUC: 0.97/0.99) and chronic pt (AUC (0.89/0.91) from the control group. |  |
| Chiba et al. (2010) |  |  |  | Significant correlation between the two CB tasks (p < 0.05). | 4 pt had significantly greater rightward deviation in the more complex task (p < 0.05). |  |
| Vossel et al. (2010) | Sensitive. |  |  | Correlation with CB latency quotient and confrontation technique was low, but significant with CoC of line and star cancellation tasks and deviation of line bisection (p < 0.001). | Distinguish between the control group, extinction pt, moderate and severe neglect. | Easily applied, reliable, quick |
| Rabuffetti et al. (2012) | Not significant (p = 0.22) but higher than PnP. |  |  | No strong correlation, never exceeded 0.30 in the module (the largest coefficient being 0.27 for correlation between mini mental state examination and number of touched distracters). | Significant differences among RUSN+ pt vs the control group and RUSN- pt (p < 0.05), significant group differences were found (p < 0.01) in 13/14 indexes. | User-friendly. |
| Bonato et al. (2013) | CB was more sensitive (detected overall left omissions (60.2%) and the PnP (6.9%). |  |  |  | Can detect neglect symptoms:  Difference L/R target detection was significant (p < 0.001).  Detection of left targets was decreasing in more complex tasks (p < 0.005). |  |
| Ulm et al. (2013) | More sensitive than NET (German BIT). |  |  |  | CB versions distinguish significantly (star cancellation p = 0.025) and line bisection (p = 0.001) between USN+ pt and the control group. Significant discriminant function was shown with (2/4) CB variables (p = 0.003). | Valid. |
| Van Kessel et al. (2013) | The computerized dual task (CVRT-D) was able to detect cases with normal BIT and computerized single-detection task (CVRT) scores. |  |  | Significant correlation coefficient for contralesional omissions for RBD ptgroup between BIT and CVRT (p < 0.05) and CVRT-D (p < 0.005). | Number of contralesional omission can distinguish RBD pt from the control group and LBD pt.  RBD pt had higher RT asymmetries (p < 0.001) compared to other groups. |  |
| Jee et al. (2015) |  |  | Inter-rater: 0.84 -0.98 (p < 0.001).  Intra-rater 0.84-0.91  (p < 0.001). |  |  | Feasible, valid and reliable. |
| Pallavicini et al. (2015) | The CB card dealing task was more sensitive than the conventional method. CB cancellation tasks were equally sensitive to PnP. |  |  |  | L side number of omissions can significantly distinguish between USN+ and USN- groups in CB cancellation tasks (p < 0.01) and card dealing task (p < 0.05). | User-friendly with high usability. |
| Vaes et al. (2015) |  |  |  |  | The (21/26) CB variables can significantly (p < 0.05) distinguish between USN+ pt and the control group. | User-friendly. |
| Chung et al. (2016) | 95% sensitivity (higher than PnP line bisection with 75%, and star cancellation with 94.4%). |  |  | The computerized table setting test (CTST) parameters significantly correlated with total neglect score (horizontal deviation, p < 0.001, selection tendency, p < 0.001, and elapsed time p = 0.007). | CTST 3/3 parameters significantly distinguished between RBD+ and LBD pt and the control group (horizontal deviation, p < 0.001; selection tendency, p < 0.001; and elapsed time, p = 0.006). | Feasible. |
| Mizuno et al. (2016) | CB may be more sensitive than BIT. |  |  |  | Circle test (average cancellation time) can distinguish between pt and controls. Visual and visuomotor test detected significant difference in average RT (p < 0.01) between stimulus-centered pt and other pt groups and control. |  |
| Ten Brink et al. (2016) |  |  |  | CB shape cancellation (number of intersections) showed a moderate correlation with neglect severity (p = 0.009). | The CB task can significantly distinguish between RBD+ and LUSN+ pt from the control group with significantly higher numbers of intersections (p < 0.003) and omissions (p < 0.001), similarly the number of intersections can significantly differentiate RUSN+ from LUSN+ pt (p = 0.09) and RUSN- pt (p = 0.001). |  |
| Stigchel and Nijboer (2017) | Sensitive for spatial bias deficits. |  |  | Strong correlation between Temporal order judgment (PSS) and with shape cancellation (omission difference) (p < 0.001), but not with line bisection (deviation score) (p = 0.08). | Temporal order judgment task (PSS) significantly detected patients with spatial deficits (p = 0.0002). |  |
| Machner et al. (2018) | The desk task was more sensitive than PnP and as sensitive as the Posner Task in detecting spatial attention bias. |  |  | The desk task (mean search duration) and the Posner task (mean RT) were highly correlated with the CBS score (p < 0.001).  Latency index of the desk task was correlated with the Posner task and with the bell’s task CoC (p < 0.001). . | Desk task: USN+ groups had longer left search duration and left target detection than the control group and USN- pt (p < 0.01). Posner: Severe USN+ pt had lower left detection rate than other groups (p < 0.001). The Posner (RT) can distinguish between the control group and all pt groups (p < 0.003). |  |
| Quinn et al. (2018) | 79% sensitivity (for stroke related visual field deficits). | Specificity: 88% (For stroke related visual field deficits). |  |  | . | Similar acceptability scores with PnP. |
| Andres et al. (2019) | CB tasks significantly revealed (p < 0.001) neglect/extinction symptoms for more cases than conventional tasks. |  |  |  | L/R omission in single and dual task can significantly reveal neglect (p < 0.001). |  |
| Morando et al. (2019) |  |  | High test-retest reliability over 0.75 for both tasks. | High Pearson correlation coefficient for repeated CB test and line bisection and Albert’s test respectively (offset: = 0.083, Rate: p = 0.094) and (uncrossed L/R: p = 0.098 / p = 0.080). |  | Feasible and valid. |
| Spreij et al. (2020) | Sensitivity: 51.5% (for LUSN+ pt) and 28.6% (for RUSN+ pt). | Specificity: 94.3% (for USN- and LUSN+ pt).  LUSN+: (85% Positive Predictive Value (PPV), 75.8% Negative Predictive Value (NPV) pt).  RUSN+: (40% PPV, 90.9% NPV). |  | Spearman correlation between the CB (average position of the road) and CBS (neglect severity) was significant (p = 0.05). | CB average position deviation and magnitude of sway distinguished USN+ pt from LUSN- pt (p < 0.001) and controls, but not RUSN+ vs USN- pt . |  |
| Ten Brink et al. (2020) | Adding targets can increase sensitivity. |  |  | Correlation between CB percentage of hits and shape cancellation omissions (p < 0.001) of deviation and line bisection (p < 0.001) and total CBS score (p < 0.001). | Increased number of target task can significantly affect HR performance (p = 0.008) and distinguish RBD+ from LBD and RBD- pt. |  |
| Villarreal et al. (2020) | Higher sensitivity than PnP. The CB detected more cases and could distinguish between groups. |  |  |  | RBD pt had significantly increased missed left target than the control group (p < 0.05) and LBD pt in both tasks. | High ecological validity. |
| Pierce et al. (2021) |  |  |  | No significant correlation was detected between manual exploration task mean horizontal position and deviation of line bisection or CoC of bells or apples cancellation. | Percentage of taps showed significant groups differences (p < 0.05), USN+ pt vs other groups had significantly different left percentage of taps (p = 0.037). | Quick. |

Andres, M., Geers, L., Marnette, S., Coyette, F., Bonato, M., Priftis, K., et al. (2019). Increased Cognitive Load Reveals Unilateral Neglect and Altitudinal Extinction in Chronic Stroke. *Journal of the International Neuropsychological Society* 25(6)**,** 644-653. doi: 10.1017/S1355617719000249.

Bonato, M., Priftis, K., Umiltà, C., and Zorzi, M. (2013). Computer-Based Attention-Demanding Testing Unveils Severe Neglect in Apparently Intact Patients. *Behavioural neurology***,** 179-181. doi: 10.3233/BEN-2012-129005.

Chiba, Y., Nishihara, K., and Haga, N. (2010). Evaluating visual bias and effect of proprioceptive feedback in unilateral neglect. *Journal of Clinical Neuroscience* 17(9)**,** 1148-1152. doi: 10.1016/j.jocn.2010.02.017.

Chung, S., Park, E., Ye, B.S., Lee, H., Chang, H.-J., Song, D., et al. (2016). The Computerized Table Setting Test for Detecting Unilateral Neglect. *PloS one* 11**,** e0147030. doi: 10.1371/journal.pone.0147030.

Deouell, L.Y., Sacher, Y., and Soroker, N. (2005). Assessment of spatial attention after brain damage with a dynamic reaction time test. *Journal of the International Neuropsychological Society* 11(6)**,** 697-707. doi: 10.1017/S1355617705050824.

Erez, A., Katz, N., Ring, H., and Soroker, N. (2009). Assessment of spatial neglect using computerised feature and conjunction visual search tasks. *Neuropsychol Rehabil* 19(5)**,** 677-695. doi: 10.1080/09602010802711160.

Jee, H., Kim, J., Kim, C., Kim, T., and Park, J. (2015). Feasibility of a Semi-computerized Line Bisection Test for Unilateral Visual Neglect Assessment. *Applied Clinical Informatics* 6(2)**,** 400-417. doi: 10.4338/aci-2015-01-ra-0002.

Liang, Y., Guest, R.M., Fairhurst, M.C., and Potter, J.M. (2007). Feature-based assessment of visuo-spatial neglect patients using hand-drawing tasks. *Pattern Analysis and Applications* 10(4)**,** 361-374. doi: 10.1007/s10044-007-0074-x.

List, A., Brooks, J.L., Esterman, M., Flevaris, A.V., Landau, A.N., Bowman, G., et al. (2008). Visual hemispatial neglect, re-assessed. *Journal of the International Neuropsychological Society* 14(2)**,** 243-256. doi: 10.1017/S1355617708080284.

Machner, B., Koenemund, I., von der Gablent, J., Bays, P.M., and Sprenger, A. (2018). The Ipsilesional Attention Bias in Right-Hemisphere Stroke Patients as Revealed by a Realistic Visual Search Task: Neuroanatomical Correlates and Functional Relevance. *Neuropsychology* 32(7)**,** 850-865. doi: 10.1037/neu0000493.

Marshall, S.C., Grinnell, D., Heisel, B., Newall, A., and Hunt, L. (1997). Attentional deficits in stroke patients: A visual dual task experiment. *Archives of Physical Medicine and Rehabilitation* 78(1)**,** 7-12. doi: 10.1016/S0003-9993(97)90002-2.

Mizuno, K., Kato, K., Tsuji, T., Shindo, K., Kobayashi, Y., and Liu, M. (2016). Spatial and temporal dynamics of visual search tasks distinguish subtypes of unilateral spatial neglect: Comparison of two cases with viewer-centered and stimulus-centered neglect. *Neuropsychological Rehabilitation* 26(4)**,** 610-634. doi: 10.1080/09602011.2015.1051547.

Morando, M., Bonotti, E., Giannarelli, G., Olivieri, S., Dellepiane, S., and Cecchi, F. (2019). "Monitoring Home-Based Activity of Stroke Patients: A Digital Solution for Visuo-Spatial Neglect Evaluation: Proceedings of the 4th International Conference on NeuroRehabilitation (ICNR2018), October 16-20, 2018, Pisa, Italy."), 696-701.

Pallavicini, F., Pedroli, E., Serino, S., Dell'Isola, A., Cipresso, P., Cisari, C., et al. (2015). Assessing Unilateral Spatial Neglect using advanced technologies: The potentiality of mobile virtual reality. *Technology and Health Care* 23(6)**,** 795-807. doi: 10.3233/THC-151039.

Pierce, J.E., Ronchi, R., Thomasson, M., Rossi, I., Casati, C., Saj, A., et al. (2021). A novel computerized assessment of manual spatial exploration in unilateral spatial neglect. *Neuropsychological Rehabilitation*. doi: 10.1080/09602011.2021.1875850.

Quinn, T.J., Livingstone, I., Weir, A., Shaw, R., Breckenridge, A., McAlpine, C., et al. (2018). Accuracy and Feasibility of an Android-Based Digital Assessment Tool for Post Stroke Visual Disorders-The StrokeVision App. *Front Neurol* 9**,** 146. doi: 10.3389/fneur.2018.00146.

Rabuffetti, M., Farina, E., Alberoni, M., Pellegatta, D., Appollonio, I., Affanni, P., et al. (2012). Spatio-temporal features of visual exploration in unilaterally brain-damaged subjects with or without neglect: Results from a touchscreen test. *PLoS ONE* 7(2). doi: 10.1371/journal.pone.0031511.

Rabuffetti, M., Ferrarin, M., Spadone, R., Pellegatta, D., Gentileschi, V., Vallar, G., et al. (2002). Touch-screen system for assessing visuo-motor exploratory skills in neuropsychological disorders of spatial cognition. *Medical and Biological Engineering and Computing* 40(6)**,** 675-686. doi: 10.1007/BF02345306.

Rengachary, J., d'Avossa, G., Sapir, A., Shulman, G.L., and Corbetta, M. (2009). Is the posner reaction time test more accurate than clinical tests in detecting left neglect in acute and chronic stroke? *Arch Phys Med Rehabil* 90(12)**,** 2081-2088. doi: 10.1016/j.apmr.2009.07.014.

Spreij, L.A., Ten Brink, A.F., Visser-Meily, J.M.A., and Nijboer, T.C.W. (2020). Simulated driving: The added value of dynamic testing in the assessment of visuo-spatial neglect after stroke. *Journal of Neuropsychology* 14(1)**,** 28-45. doi: 10.1111/jnp.12172.

Stigchel, S., and Nijboer, T. (2017). Temporal order judgements as a sensitive measure of the spatial bias in patients with visuospatial neglect. *Journal of neuropsychology* 12. doi: 10.1111/jnp.12118.

Ten Brink, A.F., Elshout, J., Nijboer, T.C., and Van der Stigchel, S. (2020). How does the number of targets affect visual search performance in visuospatial neglect? *Journal of Clinical and Experimental Neuropsychology* 42(10)**,** 1010-1027.

Ten Brink, A.F., van der Stigchel, S., Visser-Meily, J.M.A., and Nijboer, T.C.W. (2016). You never know where you are going until you know where you have been: Disorganized search after stroke. *Journal of Neuropsychology* 10(2)**,** 256-275. doi: 10.1111/jnp.12068.

Ulm, L., Wohlrapp, D., Meinzer, M., Steinicke, R., Schatz, A., Denzler, P., et al. (2013). A Circle-Monitor for Computerised Assessment of Visual Neglect in Peripersonal Space. *PLOS ONE* 8(12)**,** e82892. doi: 10.1371/journal.pone.0082892.

Vaes, N., Lafosse, C., Nys, G., Schevernels, H., Dereymaeker, L., Oostra, K., et al. (2015). Capturing peripersonal spatial neglect: An electronic method to quantify visuospatial processes. *Behavior Research Methods* 47(1)**,** 27-44.

Van Kessel, M.E., Van Nes, I.J.W., Geurts, A.C.H., Brouwer, W.H., and Fasotti, L. (2013). Visuospatial asymmetry in dual-task performance after subacute stroke. *Journal of Neuropsychology* 7(1)**,** 72-90. doi: 10.1111/j.1748-6653.2012.02036.x.

Villarreal, S., Linnavuo, M., Sepponen, R., Vuori, O., Jokinen, H., and Hietanen, M. (2020). Dual-Task in Large Perceptual Space Reveals Subclinical Hemispatial Neglect. *Journal of the International Neuropsychological Society* 26(10)**,** 993-1005. doi: 10.1017/S1355617720000508.

Vossel, S., Eschenbeck, P., Weiss, P., and Fink, G. (2010). Assessing visual extinction in right-hemisphere stroke patients with and without neglect. *Klinische Neurophysiologie. Conference* 41(1).
