# Supplementary material for: Computer-based assessment of unilateral spatial neglect: A systematic review
Source: Front Neurosci. 2022 Aug 19;16:912626. doi: 10.3389/fnins.2022.912626 (PMC9437703; doi:10.3389/fnins.2022.912626)
Supplement: Supplementary file 4 [file Table_4.DOCX]

Supplementary Material

# Supplementary Tables

# 1.4. Supplementary Table 4. List of excluded citations with reasons for exclusion.

| **List of excluded citations** | **Reasons for exclusion** |
| --- | --- |
| Anderson, B., Mennemeier, M., and Chatterjee, A. (2000). Variability not ability: Another basis for performance decrements in neglect. Neuropsychologia 38(6), 785-796. doi: 10.1016/S0028-3932(99)00137-2. | Absence or unclear evaluation of the task or comparison with conventional tool. |
| Anton, H.A., Hershler, C., Lloyd, P., and Murray, D. (1988). Visual neglect and extinction: A new test. Archives of Physical Medicine and Rehabilitation 69(12), 1013-1016. | Out of scope. |
| Arguin, M., and Bub, D. (1993). Modulation of the directional attention deficit in visual neglect by hemispatial factors. Brain and Cognition 22(2), 148-160. | Out of scope. |
| Bartolomeo, P., Chokron, S., and Sieroff, E. (1999). Facilitation instead of inhibition for repeated right-sided events in left neglect. NeuroReport: For Rapid Communication of Neuroscience Research 10(16), 3353-3357. | Absence or unclear evaluation of the task or comparison with conventional tool. |
| Baylis, G.C., Baylis, L.L., and Gore, C.L. (2004). Visual neglect can be object-based or scene-based depending on task representation. Cortex 40(2), 237-246. doi: 10.1016/S0010-9452(08)70119-9. | Absence or unclear evaluation of the task or comparison with conventional tool. |
| Behrmann, M., and Tipper, S.P. (1999). Attention Accesses Multiple Reference Frames: Evidence From Visual Neglect. Journal of Experimental Psychology: Human Perception & Performance 25(1), 83-101. | Absence or unclear evaluation of the task or comparison with conventional tool. |
| Beis, J.M., André, J.M., and Saguez, A. (1994). Detection of visual field deficits and visual neglect with computerized light emitting diodes. Archives of Physical Medicine and Rehabilitation 75(6), 711-714. doi: 10.1016/0003-9993(94)90201-1. | Absence of or unclear information regarding the design of the task (e.g., apparatus, task demands). |
| Benson, V., Ietswaart, M., and Milner, D. (2012). Eye movements and verbal report in a single case of visual neglect. PLoS ONE 7(8). | Out of scope. |
| Bergego, C., Azouvi, P., Deloche, G., Samuel, C., Louis-Dreyfus, A., Kaschel, R., et al. (1997). Rehabilitation of unilateral neglect: A controlled multiple-baseline-across-subjects trial using computerised training procedures. Neuropsychological Rehabilitation 7(4), 279-294. doi: 10.1080/713755548. | Out of scope. |
| Blini, E., Romeo, Z., Spironelli, C., Pitteri, M., Meneghello, F., Bonato, M., et al. (2016). Multi-tasking uncovers right spatial neglect and extinction in chronic left-hemisphere stroke patients. Neuropsychologia 92, 147-157. doi: 10.1016/j.neuropsychologia.2016.02.028. | Absence or unclear evaluation of the task or comparison with conventional tool. |
| Borsotti, M., Mosca, I.E., Di Lauro, F., Pancani, S., Bracali, C., Dore, T., et al. (2020). The Visual Scanning Test: a newly developed neuropsychological tool to assess and target rehabilitation of extrapersonal visual unilateral spatial neglect. Neurol Sci 41(5), 1145-1152. doi: 10.1007/s10072-019-04218-2. | Absence or unclear evaluation of the task or comparison with conventional tool. |
| Bourgeois, A., Chica, A.B., Migliaccio, R., de Schotten, M.T., and Bartolomeo, P. (2012). Cortical control of inhibition of return: Evidence from patients with inferior parietal damage and visual neglect. Neuropsychologia 50(5), 800-809. doi: 10.1016/j.neuropsychologia.2012.01.014. | Absence or unclear evaluation of the task or comparison with conventional tool. |
| Bublak, P., Finke, K., Krummenacher, J., Preger, R., Kyllingsbaek, S., Müller, H.J., et al. (2005). Usability of a theory of visual attention (TVA) for parameter-based measurement of attention II: evidence from two patients with frontal or parietal damage. J Int Neuropsychol Soc 11(7), 843-854. doi: 10.1017/s1355617705050988. | Not focusing (or clarifying focusing) on assessing the presence or severity of unilateral spatial neglect. |
| Carter, A.R., McAvoy, M.P., Siegel, J.S., Hong, X., Astafiev, S.V., Rengachary, J., et al. (2017). Differential white matter involvement associated with distinct visuospatial deficits after right hemisphere stroke. Cortex 88, 81-97. doi: 10.1016/j.cortex.2016.12.009. | Not focusing (or clarifying focusing) on assessing the presence or severity of unilateral spatial neglect. |
| Champod, A.S., Taylor, K., and Eskes, G.A. (2014). Development of a new computerized prism adaptation procedure for visuo-spatial neglect. Journal of Neuroscience Methods 235, 65-75. doi: 10.1016/j.jneumeth.2014.05.023. | Absence or unclear evaluation of the task or comparison with conventional tool. |
| Cheyne, J.A., Solman, G.J.F., Carriere, J.S.A., and Smilek, D. (2009). Anatomy of an error: A bidirectional state model of task engagement/disengagement and attention-related errors. COGNITION 111(1), 98-113. doi: 10.1016/j.cognition.2008.12.009. | Not focusing (or clarifying focusing) on assessing the presence or severity of unilateral spatial neglect. |
| Chiba, Y., Yamaguchi, A., and Eto, F. (2006). Assessment of sensory neglect: A study using moving images. Neuropsychological Rehabilitation 16(6), 641-652. doi: 10.1080/09602010543000073. | Absence or unclear evaluation of the task or comparison with conventional tool. |
| Clatworthy, P.L., Warburton, E.A., Tolhurst, D.J., and Baron, J.C. (2013). Visual contrast sensitivity deficits in 'normal' visual field of patients with homonymous visual field defects due to stroke: A pilot study. Cerebrovascular Diseases 36(5-6), 329-335. doi: 10.1159/000354810. | Not focusing (or clarifying focusing) on assessing the presence or severity of unilateral spatial neglect. |
| Cohen, A., Ivry, R.B., Rafal, R.D., and Kohn, C. (1995). Activating Response Codes by Stimuli in the Neglected Visual Field. Neuropsychology 9(2), 165-173. | Absence or unclear evaluation of the task or comparison with conventional tool. |
| Corbetta, M., Kincade, M.J., Lewis, C., Snyder, A.Z., and Sapir, A. (2005). Neural basis and recovery of spatial attention deficits in spatial neglect. Nature Neuroscience 8(11), 1603-1610. doi: 10.1038/nn1574. | Out of scope. |
| Coslett, H.B. (1997). Neglect in vision and visual imagery: a double dissociation. Brain 120 ( Pt 7), 1163-1171. doi: 10.1093/brain/120.7.1163. | Absence or unclear evaluation of the task or comparison with conventional tool. |
| Costantini, M., Bueti, D., Pazzaglia, M., and Aglioti, S.M. (2007). Temporal Dynamics of Visuo-Tactile Extinction Within and Between Hemispaces. Neuropsychology 21(2), 242-250. | Not focusing (or clarifying focusing) on assessing the presence or severity of unilateral spatial neglect. |
| Crewther, S.G., Wijesundera, C., Wijeratne, T., Kong, G., and Vingrys, A.J. (2018). High prevalence of visual field impairments in acute stroke patients. INVESTIGATIVE OPHTHALMOLOGY & VISUAL SCIENCE 59(9). | Not focusing (or clarifying focusing) on assessing the presence or severity of unilateral spatial neglect. |
| Crottaz-Herbette, S., Fornari, E., and Clarke, S.(2014). Prismatic Adaptation Changes Visuospatial Representation in the Inferior Parietal Lobule. Journal of Neuroscience 34(35), 11803-11811. doi: 10.1523/jneurosci.3184-13.2014. | Not focusing (or clarifying focusing) on assessing the presence or severity of unilateral spatial neglect. |
| Crottaz-Herbette, S., Tissieres, I., Fornari, E., Rapin, P.-A., and Clarke, S. (2019). Remodelling the attentional system after left hemispheric stroke: Effect of leftward prismatic adaptation. Cortex 115, 43-55. doi: 10.1016/j.cortex.2019.01.007. | Not focusing (or clarifying focusing) on assessing the presence or severity of unilateral spatial neglect. |
| Cullen, B., Brennan, D., Manly, T., and Evans, J.J. (2016). Towards Validation of a New Computerised Test of Goal Neglect: Preliminary Evidence from Clinical and Neuroimaging Pilot Studies. PloS one 11(1), e0148127. doi: 10.1371/journal.pone.0148127. | Not focusing (or clarifying focusing) on assessing the presence or severity of unilateral spatial neglect. |
| D'Erme, P., Robertson, I., Bartolomeo, P., Daniele, A., and Gainotti, G. (1992). Early rightwards orienting of attention on simple reaction time performance in patients with left-sided neglect. Neuropsychologia 30(11), 989-1000. doi: 10.1016/0028-3932(92)90050-V. | Not focusing (or clarifying focusing) on assessing the presence or severity of unilateral spatial neglect. |
| Dalmaijer, E.S., Van der Stigchel, S., Nijboer, T.C., Cornelissen, T.H., and Husain, M. (2015). CancellationTools: All-in-one software for administration and analysis of cancellation tasks. Behavior research methods 47(4), 1065-1075. | Absence or unclear evaluation of the task or comparison with conventional tool. |
| DeGutis, J.M., and Van Vleet, T.M. (2010). Tonic and phasic alertness training: a novel behavioral therapy to improve spatial and non-spatial attention in patients with hemispatial neglect. FRONTIERS IN HUMAN NEUROSCIENCE 4. doi: 10.3389/fnhum.2010.00060. | Not focusing (or clarifying focusing) on assessing the presence or severity of unilateral spatial neglect. |
| Dodds, C.M., van Belle, J., Peers, P.V., Dove, A., Cusack, R., Duncan, J., et al. (2008). The Effects of Time-on-Task and Concurrent Cognitive Load on Normal Visuospatial Bias. Neuropsychology 22(4), 545-552. | Out of scope. |
| Donnelly, N., Guest, R., Fairhurst, M., Potter, J., Deighton, A., and Patel, M. (1999). Developing algorithms to enhance the sensitivity of cancellation tests of visuospatial neglect. Behavior Research Methods Instruments & Computers 31(4), 668-673. doi: 10.3758/bf03200743. | Absence or unclear evaluation of the task or comparison with conventional tool. |
| Dove, M., Eskes, G., Klein, R., and Shore, D. (2007). A left attentional bias in chronic neglect: A case study using temporal order judgments. Neurocase 13(1), 37-49. doi: 10.1080/13554790601174146. | Out of scope. |
| Dunai, J., Bennett, K., Fotiades, A., Kritikos, A., and Castiello, U. (1999). Modulation of unilateral neglect as a function of direction of object motion. Neuroreport 10(5), 1041-1047. doi: 10.1097/00001756-199904060-00027. | Out of scope. |
| Egly, R., Driver, J., and Rafal, R.D. (1994). Shifting Visual Attention Between Objects and Locations: Evidence From Normal and Parietal Lesion Subjects. Journal of Experimental Psychology: General 123(2), 161-177. | Out of scope. |
| Esterman, M., McGlinchey-Berroth, R., and Milberg, W. (2000). Preattentive and Attentive Visual Search in Individuals With Hemispatial Neglect. Neuropsychology 14(4), 599-611. | Absence or unclear evaluation of the task or comparison with conventional tool. |
| Fellrath, J., and Ptak, R. (2015). The role of visual saliency for the allocation of attention: Evidence from spatial neglect and hemianopia. Neuropsychologia 73, 70-81. doi: 10.1016/j.neuropsychologia.2015.05.003. | Absence or unclear evaluation of the task or comparison with conventional tool. |
| Fimm, B., Zahn, R., Mull, M., Kemeny, S., Buchwald, F., Block, F., et al. (2001). Asymmetries of visual attention after circumscribed subcortical vascular lesions. J Neurol Neurosurg Psychiatry 71(5), 652-657. doi: 10.1136/jnnp.71.5.652. | Out of scope. |
| Fortis, P., Goederth, K.M., and Barrett, A.M. (2011). Prism adaptation differently affects motor-intentional and perceptual-attentional biases in healthy individuals. Neuropsychologia 49(9), 2718-2727. doi: 10.1016/j.neuropsychologia.2011.05.020. | Out of scope. |
| Friedrich, F.J., Egly, R., Rafal, R.D., and Beck, D. (1998). Spatial Attention Deficits in Humans: A Comparison of Superior Parietal and Temporal-Parietal Junction Lesions. Neuropsychology 12(2), 193-207. | Not focusing (or clarifying focusing) on assessing the presence or severity of unilateral spatial neglect. |
| Grabowska, A., Marchewka, A., Seniów, J., Polanowska, K., Jednoróg, K., Królicki, L., et al. (2011). Emotionally negative stimuli can overcome attentional deficits in patients with visuo-spatial hemineglect. Neuropsychologia 49(12), 3327-3337. doi: 10.1016/j.neuropsychologia.2011.08.006. | Absence or unclear evaluation of the task or comparison with conventional tool. |
| Grimsen, C., Hildebrandt, H., and Fahle, M. (2008). Dissociation of egocentric and allocentric coding of space in visual search after right middle cerebral artery stroke. Neuropsychologia 46(3), 902-914. doi: 10.1016/j.neuropsychologia.2007.11.028. | Out of scope. |
| Guest, R., Fairhurst, M., and Potter, J. (2002). Diagnosis of Visuo-Spatial Neglect Using Dynamic Sequence Features from a Cancellation Task. Pattern Analysis & Applications 5, 261-270. | Absence or unclear evaluation of the task or comparison with conventional tool. |
| Halligan, P.W., and Marshall, J.C. (1989). Two techniques for the assessment of line bisection in visuo-spatial neglect: A single case study. Journal of Neurology Neurosurgery and Psychiatry 52(11), 1300-1302. doi: 10.1136/jnnp.52.11.1300. | Absence or unclear evaluation of the task or comparison with conventional tool. |
| Habekost, T., and Bundesen, C. (2003). Patient assessment based on a theory of visual attention (TVA): Subtle deficits after a right frontal-subcortical lesion. Neuropsychologia 41(9), 1171-1188. doi: 10.1016/S0028-3932(03)00018-6. | Not focusing (or clarifying focusing) on assessing the presence or severity of unilateral spatial neglect.. |
| Halligan, P.W., and Marshall, J.C. (1989). Two techniques for the assessment of line bisection in visuo-spatial neglect: A single case study. Journal of Neurology Neurosurgery and Psychiatry 52(11), 1300-1302. doi: 10.1136/jnnp.52.11.1300. | Absence or unclear evaluation of the task or comparison with conventional tool. |
| Hill, N.J., Mooney, S.W.J., Ryklin, E.B., and Prusky, G.T. (2019). Shady: A software engine for real-time visual stimulus manipulation. Journal of neuroscience methods 320, 79-86. doi: 10.1016/j.jneumeth.2019.03.020. | Not focusing (or clarifying focusing) on assessing the presence or severity of unilateral spatial neglect.. |
| Hillis, A.E., Mordkoff, J.T., and Caramazza, A. (1999). Mechanisms of spatial attention revealed by hemispatial neglect. Cortex 35(3), 433-442. doi: 10.1016/s0010-9452(08)70811-6. | Out of scope. |
| Hillis, A.E., Rapp, B., Benzing, L., and Caramazza, A. (1998). Dissociable coordinate frames of unilateral spatial neglect: "viewer-centered" neglect. Brain Cogn 37(3), 491-526. doi: 10.1006/brcg.1998.1010. | Out of scope. |
| Hopfner, S., Kesselring, S., Cazzoli, D., Gutbrod, K., Laube-Rosenpflanzer, A., Chechlacz, M., et al. (2015). Neglect and Motion Stimuli--Insights from a Touchscreen-Based Cancellation Task. PLoS One 10(7), e0132025. doi: 10.1371/journal.pone.0132025. | Absence or unclear evaluation of the task or comparison with conventional tool. |
| Ishiai, S., Koyama, Y., Nakano, N., Seki, K., Nishida, Y., and Hayashi, K. (2004). Image of a line is not shrunk but neglected. Absence of crossover in unilateral spatial neglect. Neuropsychologia 42(2), 251-256. doi: 10.1016/s0028-3932(03)00153-2. | Not focusing (or clarifying focusing) on assessing the presence or severity of unilateral spatial neglect. |
| Kaizer, F., Korner-Bitensky, N., Mayo, N., Becker, R., and Coopersmith, H. (1988). Response time of stroke patients to a visual stimulus. Stroke 19(3), 335-339. doi: 10.1161/01.STR.19.3.335. | Absence or unclear evaluation of the task or comparison with conventional tool. |
| Kerkhoff, G., and Marquardt, C. (1995). VS - A new computer program for detailed offline analysis of visual-spatial perception. Journal of Neuroscience Methods 63(1-2), 75-84. doi: 10.1016/0165-0270(95)00090-9. | Absence or unclear evaluation of the task or comparison with conventional tool. |
| Kim, E.J., Lee, B.H., Park, K.C., Suh, M.K., Ku, B.D., Heilman, K.M., et al. (2009). Consecutive versus return motor perseveration during line cancellation task in hemispatial neglect. Cogn Behav Neurol 22(2), 122-126. doi: 10.1097/WNN.0b013e3181a7227f. | Out of scope. |
| Kim, J.H., Lee, B.H., Go, S.M., Seo, S.W., Heilman, K.M., and Na, D.L. (2015). Improvement of hemispatial neglect by a see-through head-mounted display: a preliminary study. J Neuroeng Rehabil 12, 114. doi: 10.1186/s12984-015-0094-5. | Out of scope. |
| Kurylo, D.D., Waxman, R., and Kezin, O. (2006). Spatial-temporal characteristics of perceptual organization following acquired brain injury. Brain injury 20(3), 237-244. doi: 10.1080/02699050500487415. | Not focusing (or clarifying focusing) on assessing the presence or severity of unilateral spatial neglect. |
| Làdavas, E., Paladini, R., and Cubelli, R. (1993). Implicit associative priming in a patient with left visual neglect. Neuropsychologia 31(12), 1307-1320. doi: 10.1016/0028-3932(93)90100-E. | Out of scope. |
| Laeng, B., Brennen, T., and Espeseth, T. (2002). Fast responses to neglected targets in visual search reflect pre-attentive processes: An exploration of response times in visual neglect. Neuropsychologia 40(9), 1622-1636. doi: 10.1016/S0028-3932(01)00230-5. | Absence or unclear evaluation of the task or comparison with conventional tool. |
| Lavie, N., and Robertson, I.H. (2001). The role of perceptual load in neglect: rejection of ipsilesional distractors is facilitated with higher central load. J Cogn Neurosci 13(7), 867-876. doi: 10.1162/089892901753165791. | Absence or unclear evaluation of the task or comparison with conventional tool. |
| Machado, L., and Rafal, R.D. (2004). Control of Fixation and Saccades in Humans With Chronic Lesions of Oculomotor Cortex. Neuropsychology 18(1), 115-123. | Out of scope. |
| Marshall, J.C., and Halligan, P.W. (1991). A STUDY OF PLANE BISECTION IN 4 CASES OF VISUAL NEGLECT. Cortex 27(2), 277-284. doi: 10.1016/s0010-9452(13)80132-3. | Out of scope. |
| Marin, D., Pitteri, M., Della Puppa, A., Meneghello, F., Biasutti, E., Priftis, K., et al. (2016). Mental Time Line Distortion in Right-Brain-Damaged Patients: Evidence From a Dynamic Spatiotemporal Task. Neuropsychology 30(3), 338-345. | Out of scope. |
| Mattingley, J.B., Robertson, I.H., and Driver, J. (1998). Modulation of covert visual attention by hand movement: Evidence from parietal extinction after right-hemisphere damage. Neurocase 4(3), 245-253. | Out of scope. |
| McGeorge, P., Beschin, N., and Sala, S.D. (2006). Representing Target Motion: The Role of the Right Hemisphere in the Forward Displacement Bias. Neuropsychology 20(6), 708-715. | Not focusing (or clarifying focusing) on assessing the presence or severity of unilateral spatial neglect. |
| Mondor, T.A., and Amirault, K.J. (1998). Effect of Same- and Different-Modality Spatial Cues on Auditory and Visual Target Identification. Journal of Experimental Psychology: Human Perception & Performance 24(3), 745-755. | Out of scope. |
| Nijboer, T.C.W., Olthoff, L., Van Der Stigchel, S., and Visser-Meily, J.M.A. (2014). Prism adaptation improves postural imbalance in neglect patients. NeuroReport 25(5), 307-311. doi: 10.1097/WNR.0000000000000088. | Out of scope. |
| Nogueira, R.G., Silva, G.S., Lima, F.O., Yu-Chih, Y., Fleming, C., Branco, D., et al. (2017). The FAST-ED App: A Smartphone Platform for the Field Triage of Patients With Stroke. Stroke (00392499) 48(5), 1278-1284. doi: 10.1161/STROKEAHA.116.016026. | Absence or unclear evaluation of the task or comparison with conventional tool. |
| Oliveri, M., Bisiach, E., Brighina, F., Piazza, A., La Bua, V., Buffa, D., et al. (2001). rTMS of the unaffected hemisphere transiently reduces contralesional visuospatial heineglect. Neurology 57(7), 1338-1340. | Absence or unclear evaluation of the task or comparison with conventional tool. |
| Olivers, C.N.L., and Humphreys, G.W. (2004). Spatiotemporal Segregation in Visual Search: Evidence From Parietal Lesions. Journal of Experimental Psychology: Human Perception & Performance 30(4), 667-688. | Not focusing (or clarifying focusing) on assessing the presence or severity of unilateral spatial neglect. |
| Pashler, H. (1991). Shifting Visual Attention and Selecting Motor Responses: Distinct Attentional Mechanisms. Journal of Experimental Psychology: Human Perception & Performance 17(4), 1023-1040. | Out of scope. |
| Pegna, A.J., Caldara-Schnetzer, A.S., and Khateb, A. (2008). Visual search for facial expressions of emotion is less affected in simultanagnosia. Cortex 44(1), 46-53. doi: 10.1016/j.cortex.2006.02.001. | Not focusing (or clarifying focusing) on assessing the presence or severity of unilateral spatial neglect. |
| Pizzamiglio, L., Perani, D., Cappa, S.F., Vallar, G., Paolucci, S., Grassi, F., et al. (1998). Recovery of neglect after right hemispheric damage: H215O positron emission tomographic activation study. Archives of Neurology 55(4), 561-568. doi: 10.1001/archneur.55.4.561. | Out of scope. |
| Plummer, P., Dunai, J., and Morris, M.E. (2006). Understanding the effects of moving visual stimuli on unilateral neglect following stroke. Brain and Cognition 60(2), 156-165. doi: 10.1016/j.bandc.2005.11.001. | Absence or unclear evaluation of the task or comparison with conventional tool. |
| Posner, M.I., Cohen, Y., and Rafal, R.D. (1982). Neural systems control of spatial orienting. Philos Trans R Soc Lond B Biol Sci 298(1089), 187-198. doi: 10.1098/rstb.1982.0081. | Absence or unclear evaluation of the task or comparison with conventional tool. |
| Pouget, A., and Sejnowski, T.J. (2001). Simulating a lesion in a basis function model of spatial representations: comparison with hemineglect. Psychol Rev 108(3), 653-673. doi: 10.1037/0033-295x.108.3.653. | Out of scope. |
| Ptak, R., and Schnider, A. (2010). The dorsal attention network mediates orienting toward behaviorally relevant stimuli in spatial neglect. J Neurosci 30(38), 12557-12565. doi: 10.1523/jneurosci.2722-10.2010. | Absence or unclear evaluation of the task or comparison with conventional tool. |
| Ro, T., and Beauchamp, M. (2020). Ipsilesional perceptual deficits in hemispatial neglect: Case reports. Cortex 122, 277-287. doi: 10.1016/j.cortex.2019.03.022. | Not focusing (or clarifying focusing) on assessing the presence or severity of unilateral spatial neglect. |
| Rorden, C., and Karnath, H.-O. (2010). A simple measure of neglect severity. Neuropsychologia 48(9), 2758-2763. doi: 10.1016/j.neuropsychologia.2010.04.018. | Absence or unclear evaluation of the task or comparison with conventional tool. |
| Ro, T., Cohen, A., Ivry, R.B., and Rafal, R.D. (1998). Response channel activation and the temporoparietal junction. Brain and cognition 37(3), 461-476. doi: 10.1006/brcg.1998.1008. | Out of scope. |
| Sacchetti, D.L., Goedert, K.M., Foundas, A.L., and Barrett, A. (2015). Ipsilesional neglect: Behavioral and anatomical correlates. Neuropsychology 29(2), 183-190. | Absence or unclear evaluation of the task or comparison with conventional tool. |
| Sacher, Y., Serfaty, C., Deouell, L., Sapir, A., Henik, A., and Soroker, N. (2004). Role of disengagement failure and attentional gradient in unilateral spatial neglect -- a longitudinal study. Disability & Rehabilitation 26(12), 746-755. doi: 10.1080/09638280410001704340. | Absence or unclear evaluation of the task or comparison with conventional tool. |
| Saj, A., Cojan, Y., Assal, F., and Vuilleumier, P. (2019). Prism adaptation effect on neural activity and spatial neglect depend on brain lesion site. Cortex 119, 301-311. doi: 10.1016/j.cortex.2019.04.022. | Absence or unclear evaluation of the task or comparison with conventional tool. |
| Saj, A., Cojan, Y., Vocat, R., Luauté, J., and Vuilleumier, P. (2013). Prism adaptation enhances activity of intact fronto-parietal areas in both hemispheres in neglect patients. Cortex 49(1), 107-119. doi: 10.1016/j.cortex.2011.10.009. | Out of scope. |
| Saj, A., Pierce, J., Caroli, A., Ronchi, R., Thomasson, M., and Vuilleumier, P. (2020). Rightward exogenous attentional shifts impair perceptual memory of spatial locations in patients with left unilateral spatial neglect. Cortex 122, 187-197. doi: 10.1016/j.cortex.2019.10.002. | Out of scope. |
| Saj, A., Verdon, V., Hauert, C.-A., and Vuilleumier, P. (2018). Dissociable components of spatial neglect associated with frontal and parietal lesions. Neuropsychologia 115, 60-69. doi: 10.1016/j.neuropsychologia.2018.02.021. | Out of scope. |
| Schendel, K.L., and Robertson, L.C. (2002). Using Reaction Time to Assess Patients With Unilateral Neglect and Extinction. Journal of Clinical and Experimental Neuropsychology 24(7), 941-950. doi: 10.1076/jcen.24.7.941.8390. | Absence or unclear evaluation of the task or comparison with conventional tool. |
| Shimodozono, M., Matsumoto, S., Miyata, R., Etoh, S., Tsujio, S., and Kawahira, K. (2006). Perceptual, premotor and motor factors in the performance of a delayed-reaching task by subjects with unilateral spatial neglect. Neuropsychologia 44(10), 1752-1764. doi: 10.1016/j.neuropsychologia.2006.03.012. | Out of scope. |
| Shomstein, S., Lee, J., and Behrmann, M. (2010). Top-down and bottom-up attentional guidance: Investigating the role of the dorsal and ventral parietal cortices. Experimental Brain Research 206(2), 197-208. doi: 10.1007/s00221-010-2326-z. | Not focusing (or clarifying focusing) on assessing the presence or severity of unilateral spatial neglect. |
| Smania, N., Martini, M.C., Gambina, G., Tomelleri, G., Palamara, A., Natale, E., et al. (1998). The spatial distribution of visual attention in hemineglect and extinction patients. Brain 121 ( Pt 9), 1759-1770. doi: 10.1093/brain/121.9.1759. | Out of scope. |
| Smit, M., Van der Stigchel, S., Visser-Meily, J.M.A., Kouwenhoven, M., Eijsackers, A.L.H., and Nijboer, T.C.W. (2013). The feasibility of computer-based prism adaptation to ameliorate neglect in sub-acute stroke patients admitted to a rehabilitation center. Frontiers in Human Neuroscience (JUL). doi: 10.3389/fnhum.2013.00353. | Absence or unclear evaluation of the task or comparison with conventional tool. |
| Snow, J.C., and Mattingley, J.B. (2008). Central Perceptual Load Does Not Reduce Ipsilesional Flanker Interference in Parietal Extinction. Neuropsychology 22(3), 371-382. | Out of scope. |
| Sparing, R., Thimm, M., Hesse, M.D., Küst, J., Karbe, H., and Fink, G.R. (2009). Bidirectional alterations of interhemispheric parietal balance by non-invasive cortical stimulation. Brain 132(Pt 11), 3011-3020. doi: 10.1093/brain/awp154. | Not focusing (or clarifying focusing) on assessing the presence or severity of unilateral spatial neglect. |
| Stachowiak, F.J. (1993). Micro-computers in the assessment and rehabilitation of brain-damaged patients. Technology and health care : official journal of the European Society for Engineering and Medicine 1(1), 19-43. doi: 10.3233/THC-1993-1104. | Not focusing (or clarifying focusing) on assessing the presence or severity of unilateral spatial neglect. |
| Stuss, D.T., Binns, M.A., Murphy, K.J., and Alexander, M.P. (2002). Dissociations Within the Anterior Attentional System: Effects of Task Complexity and Irrelevant Information on Reaction Time Speed and Accuracy. Neuropsychology 16(4), 500-513. | Not focusing (or clarifying focusing) on assessing the presence or severity of unilateral spatial neglect. |
| Ten Brink, A.F., van der Stigchel, S., Visser-Meily, J.M.A., and Nijboer, T.C.W. (2016). You never know where you are going until you know where you have been: Disorganized search after stroke. Journal of Neuropsychology 10(2), 256-275. doi: 10.1111/jnp.12068. | Absence or unclear evaluation of the task or comparison with conventional tool. |
| Thimm, M., Fink, G.R., Kuest, J., Karbe, H., and Sturm, W. (2006). Impact of alertness training on spatial neglect: A behavioural and fMRI study. Neuropsychologia 44(7), 1230-1246. doi: 10.1016/j.neuropsychologia.2005.09.008. | Out of scope. |
| Tipper, S.P., and Behrmann, M. (1996). Object-Centered Not Scene-Based Visual Neglect. Journal of Experimental Psychology: Human Perception & Performance 22(5), 1261-1278. | Absence of or unclear information regarding the design of the task (e.g., apparatus, task demands). |
| Toba, M.N., Rabuffetti, M., Duret, C., Pradat-Diehl, P., Gainotti, G., and Bartolomeo, P. (2018). Component deficits of visual neglect: "Magnetic" attraction of attention vs. impaired spatial working memory. Neuropsychologia 109, 52-62. doi: 10.1016/j.neuropsychologia.2017.11.034. | Out of scope. |
| Tonin, L., Pitteri, M., Leeb, R., Zhang, H., Menegatti, E., Piccione, F., et al. (2017). Behavioral and Cortical Effects during Attention Driven Brain-Computer Interface Operations in Spatial Neglect: A Feasibility Case Study. Frontiers in Human Neuroscience 11. doi: 10.3389/fnhum.2017.00336. | Absence or unclear evaluation of the task or comparison with conventional tool. |
| Treccani, B., Cubelli, R., Sellaro, R., Umiltà, C., and Della Sala, S. (2012). Dissociation between awareness and spatial coding: evidence from unilateral neglect. J Cogn Neurosci 24(4), 854-867. doi: 10.1162/jocn_a_00185. | Absence or unclear evaluation of the task or comparison with conventional tool. |
| Trombini, M., Vestito, L., Morando, M., Mori, L., Trompetto, C., Bandini, F., et al. (2020). "Unilateral spatial neglect rehabilitation supported by a digital solution: two case-studies," in 42nd Annual International Conferences of the Ieee Engineering in Medicine and Biology Society: Enabling Innovative Technologies for Global Healthcare Embc'20.), 3670-3675. | Out of scope. |
| Vaes, N., Lafosse, C., Hemelsoet, D., Tichelt, E.V., Oostra, K., and Vingerhoets, G. (2015). Contraversive neglect? A modulation of visuospatial neglect in association with contraversive pushing. Neuropsychology 29(6), 988-997. doi: 10.1037/neu0000205. | Absence or unclear evaluation of the task or comparison with conventional tool. |
| Van der Stoep, N., Visser-Meily, J.M.A., Kappelle, L.J., de Kort, P.L.M., Huisman, K.D., Eijsackers, A.L.H., et al. (2013). Exploring near and far regions of space: Distance-specific visuospatial neglect after stroke. Journal of Clinical and Experimental Neuropsychology 35(8), 799-811. doi: 10.1080/13803395.2013.824555. | Absence or unclear evaluation of the task or comparison with conventional tool. |
| Van Vleet, T., and Robertson, L. (2009). Implicit representation and explicit detection of features in patients with hemispatial neglect. Brain : a journal of neurology 132, 1889-1897. doi: 10.1093/brain/awp109. | Absence or unclear evaluation of the task or comparison with conventional tool. |
| Verfaellie, M., Milberg, W.P., McGlinchey-Berroth, R., and Grande, L. (1995). Comparison of Cross-Field Matching and Forced-Choice Identification in Hemispatial Neglect. Neuropsychology 9(4), 427-434. | Out of scope. |
| Vossel, S., Eschenbeck, P., Weiss, P., and Fink, G. (2010). Assessing visual extinction in right-hemisphere stroke patients with and without neglect. Klinische Neurophysiologie. Conference 41(1). | Out of scope. |
| Webster, J.S., McFarland, P.T., Rapport, L.J., Morrill, B., Roades, L.A., and Abadee, P.S. (2001). Computer-assisted training for improving wheelchair mobility in unilateral neglect patients. Arch Phys Med Rehabil 82(6), 769-775. doi: 10.1053/apmr.2001.23201. | Out of scope. |
| Williamson, J.B., Haque, S., Burtis, B., Harciarek, M., Lamb, D., Zilli, E., et al. (2014). The influence of stimulus proximity on judgments of spatial relationships in patients with chronic unilateral right or left hemisphere stroke. Journal of Clinical and Experimental Neuropsychology 36(8), 787-793. doi: 10.1080/13803395.2014.940855. | Absence or unclear evaluation of the task or comparison with conventional tool. |
| Zimmermann, P., and Fimm, B. (2002). "A test battery for attentional performance."), 110-151. | Absence or unclear evaluation of the task or comparison with conventional tool. |
